# Chromatin segmentation based on a probabilistic model for read counts explains a large portion of the epigenome

**DOI:** 10.1186/s13059-015-0708-z

**Published:** 2015-07-24

**Authors:** Alessandro Mammana, Ho-Ryun Chung

**Affiliations:** Otto-Warburg-Laboratory, Epigenomics, Max Planck Institute for Molecular Genetics, D-14195 Berlin, Germany

## Abstract

Chromatin immunoprecipitation followed by sequencing (ChIP-seq) is an increasingly common experimental approach to generate genome-wide maps of histone modifications and to dissect the complexity of the epigenome. Here, we propose EpiCSeg: a novel algorithm that combines several histone modification maps for the segmentation and characterization of cell-type specific epigenomic landscapes. By using an accurate probabilistic model for the read counts, EpiCSeg provides a useful annotation for a considerably larger portion of the genome, shows a stronger association with validation data, and yields more consistent predictions across replicate experiments when compared to existing methods.

The software is available at http://github.com/lamortenera/epicseg

**Electronic supplementary material:**

The online version of this article (doi:10.1186/s13059-015-0708-z) contains supplementary material, which is available to authorized users.

## Background

A central question in biology is how cells of a multicellular organism with essentially the same genotype can establish and maintain distinct phenotypes. The four core histones H2A, H2B, H3, and H4 together with approximately 147 base pairs of DNA form the nucleosome – the fundamental repeating unit of the chromatin. These histones can be covalently modified and it is thought that these modifications carry information about the past and current cellular state [1]. Together with DNA methylation these histone modifications constitute the cell-type specific epigenome. Because the genome cannot be associated to the cell-to-cell variability, current research is focused on the epigenome.

Today many consortia, such as NIH Roadmap Epigenomics, ENCODE, Blueprint, DEEP, and IHEC [2–6], are providing genome-wide maps of histone modification generated with an experimental technique called Chromatin Immunoprecipitation followed by Sequencing (ChIP-seq [7]). Typically, for a given cell type, a panel of histone modifications are profiled in order to gain insight into the cell-type specific epigenome. This huge amount of available data calls for the development of integrative computational approaches to identify the most important, biologically meaningful features and to capture recurrent patterns.

The segmentation of epigenomes into chromatin states collapses the ChIP-seq tracks and provides an abstract view on the multi-dimensional data. A chromatin state is a recurrent pattern in the abundances of a given set of histone modifications, possibly related to a particular biological function. Chromatin segmentation aims at explaining the observed epigenomic data as a long sequence of a small number of hidden chromatin states. The idea of chromatin segmentation is not new [8–11], however, the small number of available computational tools for this task and the growing importance of epigenomic datasets suggest that there are still ample margins for improvement. Two popular tools are ChromHMM [9] and Segway [11]. In both approaches, the ChIP-seq experiments are transformed into genome-wide multivariate signals and subsequently used as observed variables in a probabilistic inference algorithm.

In ChromHMM the raw reads are assigned to non-overlapping bins of 200 bps and a sample-specific threshold is used to transform the count data to binary values. Given a hidden state, the binary vectors are modeled as independent Bernoulli random variables. This approach has some limitations. (1) There is a considerable loss of information when transforming a read count into a binary value, as the possibility of distinguishing between different levels of activity is precluded. This limitation is especially important for more recent, higher coverage ChIP-seq experiments. (2) There is no obvious way of deciding which threshold to use, despite it being critical for the final segmentation. (3) The independent Bernoulli model assumes independence between the chromatin marks given a hidden state. That would imply, for instance, that in those regions where a promoter states occurs, the presence of the mark H3K4me3 is independent from the presence of the mark H3K27ac, which is in contrast to our observations (see Results and Discussion, first paragraph). (4) A large portion of the genome is assigned to a state with no clear role, apart from being associated to read counts below the discretization threshold.

Segway works at a single base-pair resolution and transforms the counts into real values. Given a hidden state, a vector of transformed read counts is modeled as independent Gaussian random variables. The following shortcomings can be noted: (1) as in ChromHMM, the independence assumption between marks seems inadequate; (2) the choice of the monotone function is not easy to justify, especially because the resulting zero-inflated distribution can be very different from a Gaussian distribution; (3) because it works at a single base pair level, this method is orders of magnitude slower than ChromHMM, which severely limits its applicability.

In order to address these shortcomings we developed Epigenome Count-based Segmentation (EpiCSeg), a segmentation algorithm with the following main features. (1) Raw read counts can be directly used as observation symbols, thus eliminating the need for preprocessing steps. (2) An accurate discrete multivariate probability distribution is used for modeling the count vectors given a hidden state, which can recapitulate the overdispersion and correlation features observed in the data. (3) The probabilistic framework and computational efficiency are similar to those of ChromHMM, making EpiCSeg useful also for large genomes, such as the human genome.

In this article we will first give an overview of EpiCSeg and highlight its advantages relative to ChromHMM when applied to four different datasets and finally we will summarize the salient features and the most important conclusions.

## Results and discussion

### A multivariate probabilistic model for read counts

Modeling the raw count data is a considerable challenge for two reasons. First, because the number of mapped reads in a given region is overdispersed, that is, the variance across replicate experiments is so large that a simple Poisson model cannot account for it [12]. This degree of variation is especially important when modeling the variability in the read counts associated to the same chromatin state. Second, because the read abundances along the genome tend to be correlated. This can happen because of technical or biological biases, such as mappability, chromatin accessibility, and unspecific antibody binding [13], but it can also be a reflection of the biological processes taking place on the chromatin fiber. The histone modification abundances at promoters, for instance, have been shown to accurately predict the expression levels of genes [14]. Therefore it should be expected that in a given chromatin state the mark abundances vary more or less in proportion to the activity of the biological process they are related to.

**Fig. 1:**
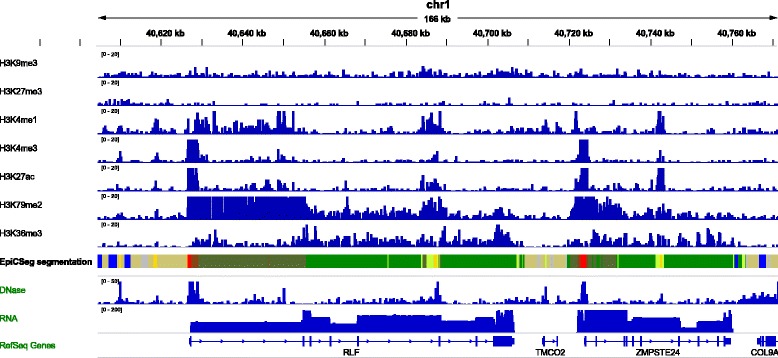
EpiCSeg segmentation in a genome browser. The first seven tracks represent the input data, that is, read counts for each genomic bin and for a panel of histone marks. The eighth track represents the main output of the algorithm, that is, a partition of the genome into segments of different types, where each type is a chromatin state and it is represented by a specific color in this picture. The last three tracks show data used for validation. It can be seen that chromatin states can be easily related to biological processes, for instance the red state represents active promoter and the green states are related to transcription

EpiCSeg’s main feature is the multivariate modeling of read counts from several histone marks, which is then integrated in a Hidden Markov Model (HMM) to produce a segmentation of the genome. The input of the algorithm is a desired number of states and a matrix where the rows represent bins (non-overlapping genomic regions of a fixed size), the columns represent histone marks, and each element contains the number of reads of a certain mark in a certain bin. The main output of the algorithm is a vector that assigns each genomic bin to one of the states (see Fig. 1 and Methods).

EpiCSeg uses a negative multinomial distribution to model the count vectors generated from each chromatin state (see Methods). This distribution, which was previously used in bioinformatics for modeling footprints in DNase I hypersensitivity data [15], has been chosen for three main reasons. Let ***X*** = (*X*_1_, *X*_2_, … *X*_*n*_) be a random count vector distributed as a negative multinomial distribution, where each component of the vector represents a different histone mark. It can be shown that the marginal distribution of each histone mark *X*_*i*_ is overdispersed, that each pair of histone marks is positively correlated, and that the negative multinomial model is more general than the independent Poisson model.

We found that this model can leverage the statistical properties observed in the experimental data to better characterize the patterns of histone mark abundances.

### Method comparison

As the chromatin segmentation problem is an unsupervised learning problem there is no clear performance score which can be used to compare segmentations by different methods. To make the comparison as fair and comprehensive as possible we adopted two strategies. First, we define and compute a number of performance indicators. These are based on the association between chromatin states and validation data or on the robustness of the segmentation algorithms. Second, we compare the different segmentations qualitatively, that is, without using any performance indicator. These comparisons also suggest alternative solutions to the task of interpreting the models provided by a segmentation algorithm. The sensitivity and specificity scores used in the quantitative comparison show how validation data can be used to identify a state which most likely represents a given genomic feature. The genome-wide statistics used in the qualitative comparison show how each state has a peculiar distribution with respect to genes and a particular signature in terms of histone mark abundances which can be related to known biological processes.

The tools chosen for the comparisons are EpiCSeg and ChromHMM. Segway could not be included here because the time required for its training process is orders of magnitude larger and also because it works at single base pair resolution, while EpiCSeg and ChromHMM, as well as our validation procedure, use a binning scheme to reduce the high noise levels in the read counts. In Additional file 1: Section 5.3, we propose a comparison with a more limited scope where we make Segway’s segmentation compatible to our binning scheme. Within the limits of this comparison and based on our performance indicators, Segway does not seem to perform as well as the other two algorithms.

To be able to draw relatively general conclusions, we compared the algorithms on four different datasets provided by the ENCODE consortium [3]:

1. IMR90: lung fibroblast cells with 27 histone marks,
2. H1: embryonic stem cells with 26 histone marks,
3. K562_1: myelogenous leukemia with 11 histone marks and one control experiment,
4. K562_2: same as above. The K562_1 and K562_2 datasets derive from an ENCODE dataset where two replicates per histone mark are available.

For each of these cell types ENCODE also provided RNA-seq and DNase I hypersensitivity experiments that were used for validation, as described in the next section.

### Quantitative comparison

We ran ChromHMM and EpiCSeg genome-wide on the four datasets. The number of chromatin states was set to 10, the number of processing threads was set to 10, and all other parameters were set to their default values. In particular, both EpiCSeg and ChromHMM use the same binning scheme. The runtime of the two algorithms were similar: both tools performed genome-wide training and prediction in 15 to 30 min with neither method showing consistently shorter runtimes (see Additional file 1: Figure S4).

**Fig. 2:**
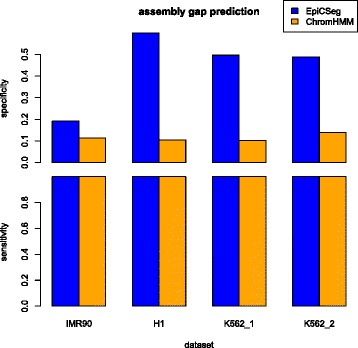
Assembly gap prediction. Chromatin states can be used as binary classifiers for detecting assembly gaps. Given a state, the true positives are the bins annotated with that state and where the reference sequence is not known (so no reads can be mapped). The specificity, or precision, is the number of true positives divided by the number of bins annotated with the state, the sensitivity is the number of true positives divided by the number of bins corresponding to assembly gaps. The state which gives maximum precision has been chosen

We first measured how well an algorithm can recognize large regions with unusually low levels of histone marks. These regions are typical in genome-wide datasets due to mappability artifacts or low levels of chromatin accessibility, and it is desirable to characterize them as precisely as possible in order not to filter out a too large portion of the genome. In this measurement, we identified the bins corresponding to assembly gaps, that is, large regions of the reference genome where the sequence is not known and where no reads can be mapped. Next, we identified which state most likely represents assembly gaps by selecting the state with the highest precision. Given a state, the precision (or specificity) is the fraction of bins assigned to this state that are assembly gaps and the sensitivity is the fraction of assembly gaps that are assigned to this state. Figure 2 shows that in all datasets and both in EpiCSeg and in ChromHMM almost all assembly gaps are annotated with the same state, however in EpiCSeg this state overlaps with the assembly gaps much more precisely, especially in the K562_1 and K562_2 datasets. The fact that the precision always remains relatively low suggests that assembly gaps are not the only regions with unusually low levels of histone marks. Assembly gaps bins were excluded in the computation of all other performance indicators.

**Fig. 3:**
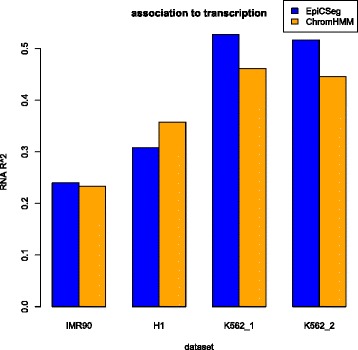
Chromatin states-based prediction of transcription levels. The value to be predicted is the log-transformed RNA-seq coverage per bin and the predictor is the chromatin state per bin. The R2 values were computed using standard linear regression

Next, we measured how well chromatin states can predict gene expression. For that purpose we used a cell-type specific RNA-seq experiment for each dataset. As a measure of gene expression levels we used the logarithm of the average RNA-seq coverage per bin (adding a pseudo-count of 1) and as a measure for predictive power we computed the R^2^ resulting from standard linear regression with a categorical predictor (the chromatin states). Figure 3 shows that EpiCSeg and ChromHMM have a similar predictive power, but the former tends to perform better. The low R^2^ values observed in the IMR90 and H1 datasets might suggest that in datasets with many ChIP-seq tracks the segmentation algorithms are less influenced by transcription-associated histone marks (for example, H3K36me3).

For the next performance indicators we used the gene annotation from GENCODE [16] and cell-type specific RNA-seq and DNase I HS experiments to characterize four different chromatin environments per cell line: RNA, characterized by a high RNA-seq signal; DNase+TSS and DNase-TSS, characterized by a high DNase I HS signal and separated according to their proximity to an annotated TSS; and intergenic, characterized by their long distance from RNA and DNase environments. We will refer to this annotation as the supervised annotation (for more details, see Methods). Note that in this annotation some bins remain unannotated and they are not considered in the following.

**Fig. 4:**
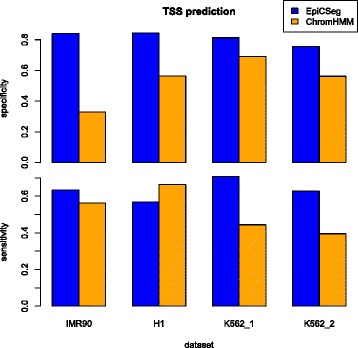
TSS prediction. Chromatin states can be used as binary classifiers for detecting transcription start sites (TSSs). Given a state, the true positives are the bins annotated with that state, with high levels of DNase hypersensitivity and where a known TSS has been annotated. The specificity, or precision, is the number of true positives divided by the number of bins annotated with the state; the sensitivity is the number of true positives divided by the number of bins corresponding to assembly gaps. The state which gives maximum precision has been chosen

Using the DNase+TSS bins as a gold-standard set of active TSSs we measured how well an algorithm can recognize active promoters. We selected the chromatin state that overlaps DNase+TSS bins with the highest precision. Figure 4 shows that EpiCSeg always identifies a chromatin state overlapping putative TSSs with a considerably higher precision than in ChromHMM. Often this state also overlaps more TSSs than in ChromHMM except in the H1 dataset (here, however, we could achieve a higher performance than with ChromHMM by merging two EpiCSeg states).

**Fig. 5:**
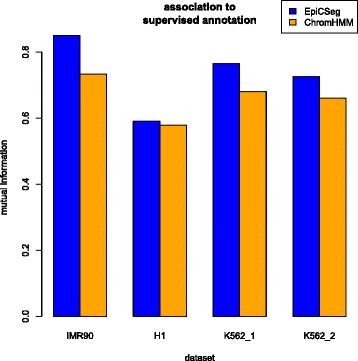
Association between the unsupervised segmentation and the supervised annotation. The manual annotation was produced using RNA-seq data, DNase I hypersensitivity data, and a gene annotation, while the chromatin states were computed using only histone mark

Next, we used the supervised annotation to compute an overall association score between external datasets (TSS annotation, RNA-seq, and DNase I hypersensitivity) and chromatin states. As performance measure we used mutual information, which can be estimated from a contingency table between the chromatin states vector and the chromatin environments vector (see Additional file 1: Section 5.2, for more details and for an alternative score). Figure 5 summarizes the results and suggests that EpiCSeg is more strongly associated to the validation data.

To test the generality of our conclusions, we repeated the comparisons described above varying the number of states from 2 to 40 (see Additional file 1: Figure S5). The results suggest that our conclusions are unlikely to depend on a particular choice of the parameters or on a particular initialization of the maximization algorithms. The results also show that the specificity in assembly gap and TSS prediction, as well as the association to transcription and to the supervised annotation, tend to grow with the number of states, while the sensitivity in assembly gap and TSS prediction decreases, suggesting that the state representing a given genomic feature will eventually be split into two or more subtypes when increasing the number of states. A large number of states, however, renders the biological interpretation of the model difficult. The BIC and AIC methods [17], which determine the optimal number of states by penalizing the likelihood according to the number of parameters, failed in suggesting a number within the explored range (data not shown). We believe that such a choice should be a compromise between interpretability and accuracy of the model.

**Fig. 6:**
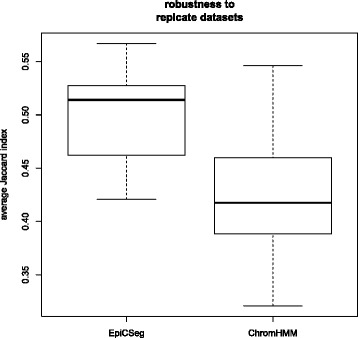
Algorithms robustness to replicate datasets. The K562_1 and the K562_2 datasets can be considered two perturbations of the same dataset. The segmentation algorithms were run independently on each chromosome on both datasets and the similarity between corresponding segmentations was measured. A higher Jaccard index means a greater robustness. The box plots show, among other things, the median (the thick line) and the first and third quartiles (the boundaries of the box) of the score distribution for each algorithm

Finally, we set out to test the algorithms’ robustness to perturbations of the input data. For assessing the robustness of a pair of segmentations we used the average Jaccard index (see Methods), which is a score between zero (completely different segmentations) and one (identical segmentations). The purpose of the first assessment is to test to which extent the chromatin states are influenced by technical variability, which includes sampling noise and differences in sequencing coverage. In fact this technical variability might affect EpiCSeg more than ChromHMM, as the former uses raw count data, while the latter uses normalized binary variables. The K562_1 and K562_2 datasets are suitable for this purpose because all samples come from the same cell type and replicate pairs are strongly correlated, even though there are considerable differences in sequencing coverage (see Additional file 1: Section 5.4). In order to have several measurements, we ran the segmentation algorithms (training and prediction) on each chromosome and each dataset separately and we computed the similarity between corresponding segmentations. The box plot in Fig. 6 shows that the segmentations obtained with EpiCSeg tend to be more consistent across replicate datasets than those obtained with ChromHMM. In both tools, the highest error rates tend to occur in those bins with a total read count across marks between 10 and 100 (see Additional file 1: Section 5.5).

**Fig. 7:**
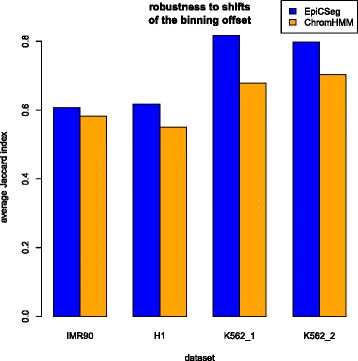
Algorithms robustness to shifts of the binning offset. Reads have been counted using two different binning schemes, both with a bin size of 200 base pairs. In the first scheme, for each chromosome, the first base of the chromosome is also the first base of the first bin. In the second scheme the bins have been shifted by 100 base pairs. The segmentation algorithms have been run using both schemes and the similarity between segmentations has been measured

In the second assessment, we test the robustness of the algorithms to changes in the binning scheme. By default both algorithms (EpiCSeg and ChromHMM), bin the genome by assigning the first 200 base pairs of each chromosome to a bin, the second 200 base pairs to the next, and so on. Here, we studied to which extent the state assignment per base pair changes after shifting all bins by 100 base pairs. Figure 7 shows that, for instance, in the K562_1 dataset with the EpiCSeg algorithm, on average more than 80 % of the base pairs annotated with a certain state in one segmentation are annotated with the corresponding state also in the segmentation that uses the alternative binning scheme. Note, however, that a certain portion of this disagreement is simply due to the fact that the boundaries of two matching segments will necessarily differ by at least 100 base pairs. If we consider the difference between the parameters of the two models learnt by EpiCSeg, the agreement seems much more convincing (see Additional file 1: Section 5.6). These results suggest that both algorithms are relatively robust to changes in the binning scheme, and that EpiCSeg tends to be more robust.

### Qualitative comparison

**Fig. 8:**
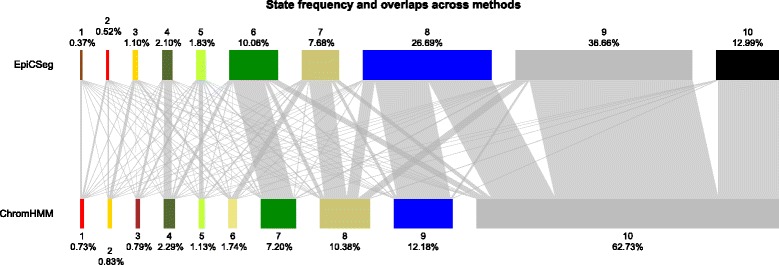
State abundance and state overlap in the 562_1 dataset. The plot shows the frequency of each state in each segmentation as bars with variable widths. Additionally the edges widths show the overlap between states of different segmentations

**Fig. 9:**
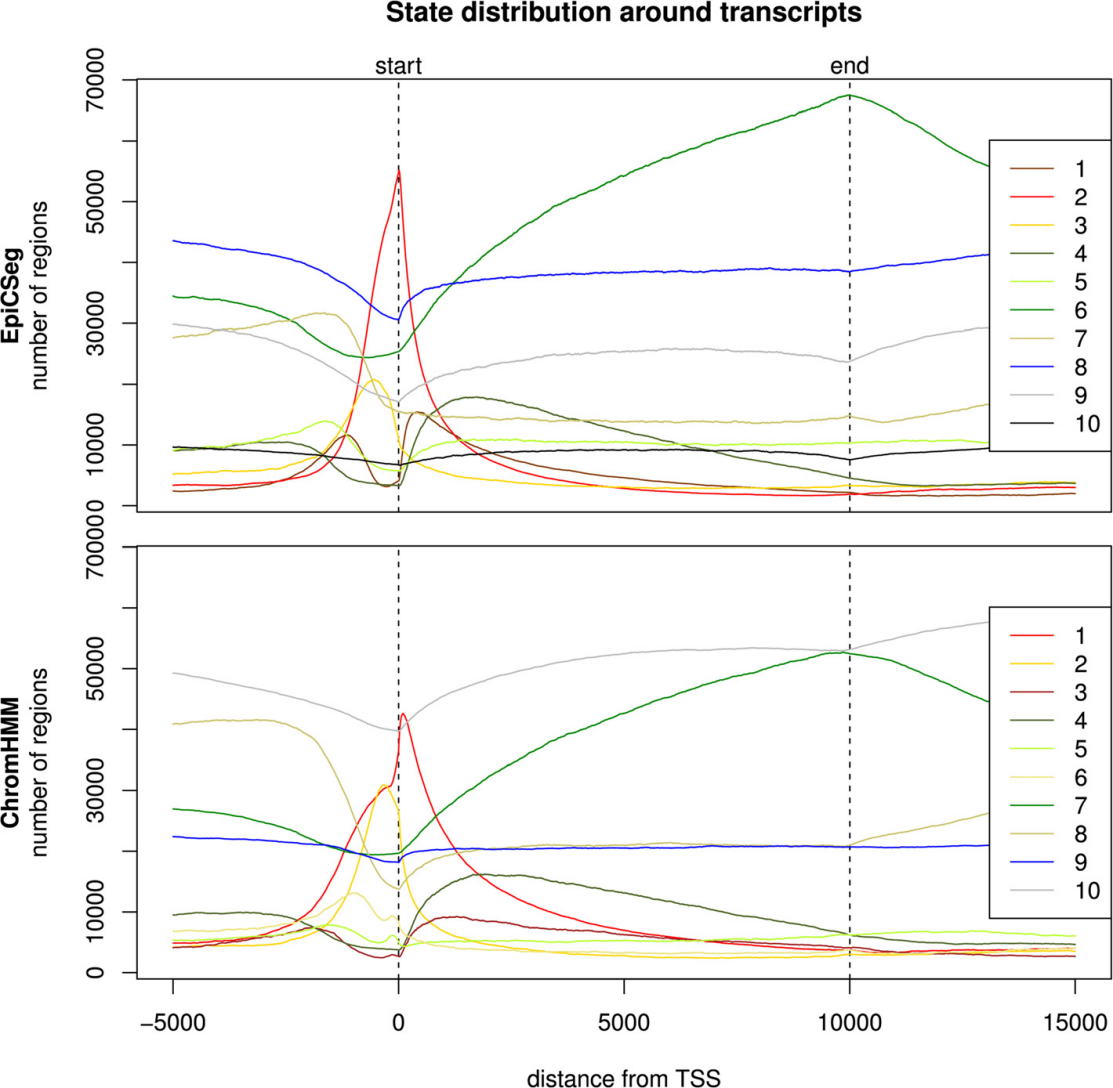
State distribution around the average transcript in the K562_1 dataset. The plot shows how often a particular state occurs at a particular position of the transcript. In each transcript, the state sequence between TSS and TES has been stretched or shrunk proportionally so that all transcripts have the same length

**Fig. 10:**
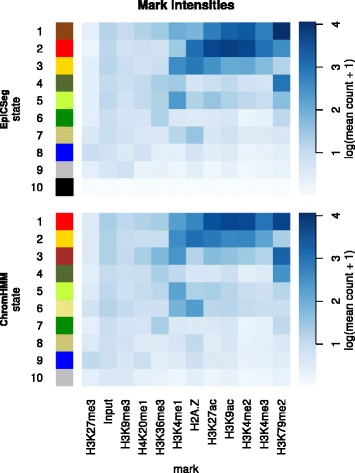
Average mark intensities per state in the K562_1 dataset. The heatmaps show the average level of a certain mark in the bins annotated with a certain state. For display purposes, the averages have been log-transformed after adding a pseudocount of 1.

In order to show the salient differences between the two algorithms without focusing on single regions, we collapsed the segmentation data into genome-wide summary statistics. The first summary statistic (Fig. 8) is a bar plot where each bar corresponds to a chromatin state and where its length is proportional to the state frequency. Additionally edges between states of the two segmentations have been drawn with widths proportional to the number of overlapping bins. Another statistic (Fig. 9) shows where each state tends to localize with respect to genes. More precisely, for each annotated transcript in the GENCODE database [16] and for a given segmentation we considered a region comprising the transcript, 5,000 bps upstream the TSS and 5,000 bps downstream the TES, we labeled each base pair with its inferred state, and we rescaled the region between TSS and TES to a reference length. Finally, taking into account all transcripts, we counted how many regions are annotated with a given state at a given position. The third summary statistic (Fig. 10) is a heatmap showing the log-transformed average histone modification levels per state.

From Fig. 9 we notice that both segmentations on the K562_1 dataset are strongly dependent on the genomic context, that is, they can capture and represent the most important biological processes acting on the chromatin. The clearest signals are a state peaking exactly at the TSS of the genes and a state which appears mainly in the body of the transcripts and peaks at the TES.

However, there are also some differences. The most apparent is that in the ChromHMM segmentation there is a state accounting for more than half of all bins, while the state distribution in EpiCSeg’s segmentation is more balanced (see Fig. 8). This background state in ChromHMM is likely to be an artifact of the discretization step and correspond to bins where most of the read counts are below the discretization threshold rather than to represent a well-defined chromatin state. The same background state mainly corresponds to three EpiCSeg states. One of them is associated to very low read counts for all marks. The analysis in Fig. 2 showed that almost all assembly gaps are annotated with this state and that they make up almost half of it. The other two states correspond to repressive chromatin environments enriched, respectively, with H3K27me3 and H3K9me3 (see Fig. 10). The second apparent difference is that that the promoter state in the EpiCSeg segmentation (state 2) is more tightly centered on the TSS, which is also reflected in the higher classification score observed in the performance comparison (Fig. 4). These conclusions are also confirmed in the other three datasets (see Performance Comparison and Additional file 1: Section 5.7, where the genome-wide segmentation statistics are shown for all other datasets).

Other smaller differences can be observed in Fig. 6. For instance, EpiCSeg separates promoter-proximal regions into those with the known set of promoter-associated marks, H3K27ac, H3K9ac, H3K4me2-3 (state 2), and those with lower levels of promoter-associated marks and a very high level of H3K79me2 (state 1), whereas ChromHMM does not make this distinction (state 1). Furthermore, the state in ChromHMM with the highest levels of H3K4me1 (state 2, probably representing enhancer regions) is very similar to the promoter state (state 1) considering its localization (Fig. 9) and marks intensities (Fig. 10), while in EpiCSeg there is a greater separation (between state 3 and state 2). These two last differences, however, cannot be always generalized to the other datasets.

### Uncertainty in state assignments

**Fig. 11:**
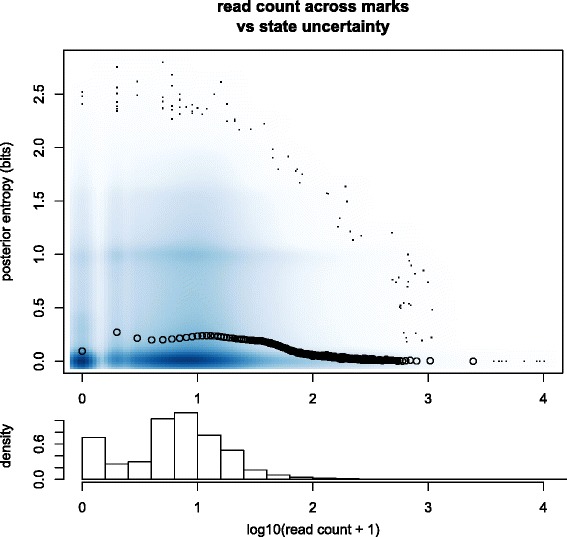
Uncertainty in the state assignments in the K562_1 dataset. The smoothed scatterplot consists in the blue shades, which show the density of points for each combination of read coverage and uncertainty, and the small black dots, which are the outliers. Additionally, entropies have been averaged over groups of bins with the same or a similar read count, so that each group consists of at least 500 bins. The empty black circles show the average entropy level per group

We explored how confidently EpiCSeg’s probabilistic model can assign a bin to a chromatin state in relation to the read coverage in the bin. As a measure of uncertainty in the state assignment we computed the posterior entropy per bin, which is the entropy of the probability distribution describing how probable each state is for that bin. The read coverage per bin is the sum of the read counts across all histone marks. The results of this explorative analysis can be seen in Fig. 11 for the K562_1 dataset, which shows: (1) a smoothed scatterplot of the posterior entropies versus the read coverage; and (2) the mean posterior entropy per read coverage level. The most apparent trend is that most of the entropies tend to cluster around 0, or to a much smaller extent, around 1, suggesting that for most of the bins the probabilistic model is very certain of the state assignment, or it is undecided between two alternatives. The second apparent trend is that the bins that can be most confidently classified are either bins with no reads at all, typically corresponding to assembly gaps, or bins with a very large number of reads, typically located in promoter regions, while bins with a read coverage between 10 and 100 are harder to classify. The same analysis performed in the other datasets leads to similar conclusions (data not shown). To summarize, EpiCSeg’s model tends to be very certain of state assignments, with a weak dependence on the read coverage. This suggests that modeling the read counts directly does not necessarily introduce a high level of uncertainty in the state inference for bins with low-counts and supports our claim that EpiCSeg allows for assigning chromatin states to a larger portion of the epigenome compared to existing approaches.

## Conclusions

We developed EpiCSeg: a new tool for segmenting the genome and determining the most important chromatin states by analyzing several ChIP-seq experiments simultaneously. Similarly to the ChromHMM algorithm, EpiCSeg divides the genome into consecutive bins and assumes a Hidden Markov Model to learn and infer the hidden sequence of chromatin states. In contrast to its predecessor, EpiCSeg’s input data are natural numbers instead of binary variables, which has two important practical advantages. First, no arbitrary thresholds on the read counts are needed to decide when a mark is present or not, as the read counts can be directly used as input data. Second, because the input data contains more information than the binary variables, EpiCSeg segmentation has the potential of being more accurate and robust.

Extensive comparisons across diverse datasets have shown that indeed the count-based segmentation can characterize active TSSs and regions with unusually low counts, distinguish between different degrees of transcription and recapitulate the validation data more precisely than the segmentation based on binary values. Moreover, this increased accuracy is also associated to an increased robustness. A qualitative analysis of EpiCSeg’s results has shown that ChromHMM’s background state, typically accounting for more than half of the genome, corresponds to at least three biologically distinct chromatin states. Finally, by modeling the read counts our method provides a starting point to introduce also other feature types such as whole genome bisulfite sequencing and DNAse hypersensitivity data to obtain a chromatin segmentation based on all commonly used epigenomic data types.

## Methods

### From reads to counts

A unique feature of EpiCSeg is that the input data can be derived from the mapped reads directly, almost without any preprocessing. The genomic regions of interest, which can be whole chromosomes, or better yet, only assembled and mappable regions, are partitioned into non-overlapping subregions of the same size called bins (in all of our analyses we used a bin size of 200 base pairs). In all our analyses, except when studying the robustness to shifts of the binning offset, the first bin of each chromosome starts at the first base pair of the reference sequence for that chromosome. To count each read into one bin the average fragment length of each ChIP-seq library needs to be taken into account, because proteins tend to bind to the middle of DNA fragments, while the reads come from the extremities of these fragments. We used NucHunter [18] to infer the average fragment length automatically from the reads. Next, we consider the corrected position of each read as its 5′ end mapping coordinate shifted in the 5′-to-3′ direction by roughly half of the average fragment length. In cases where a simpler analysis is desired, because the inferred average fragment lengths lie almost always between 100 and 200 base pairs, EpiCSeg applies a default shift of 75 base pairs. Read counting with a shift is performed using the R Bioconductor package bamsignals [19]. Counting the reads in all bins for all marks yields a count matrix for each region. These are the input to the core segmentation algorithm.

### The hidden Markov model

A hidden Markov model is used to model the count matrices and to derive a segmentation (similarly as in ChromHMM). In this framework, the *i* -th sequence of observations is encoded in the count matrix *C*^(*i*)^, and the *b* -th observation symbol in the *i* -th sequence is the *b* -th row in *C*^(*i*)^. The main idea behind the model is that there is a small number *k* of hidden states (which is an input parameter of the algorithm), and that each observation vector corresponds to a hidden state. The observation vectors and the transitions from a hidden state to the next are assumed to follow a state-dependent probability distribution (called, respectively, emission probabilities and transition probabilities), and the first hidden state in each sequence follows another probability distribution (initial probabilities).

Given this model, the algorithm does the following:

1. Initializes the emission, transition, and initial probabilities.
2. Fits the emission, transition, and initial probabilities using the Baum-Welch algorithm.
3. Infers the sequence of hidden states, which is the final segmentation, using the Viterbi or the posterior decoding algorithm (in all our analyses we use the second choice).

EpiCSeg differs from ChromHMM and from other approaches mainly by its choice of the emission probabilities, which is described in the next section. How the parameters are initialized and other implementation details are described in Additional file 1: Section 2 and 3.

### The Negative multinomial distribution

The observation vectors generated from a given hidden state are assumed to follow a negative multinomial distribution. In formulas ***X*** = (*X*_1_, *X*_2_, … *X*_*n*_) ∼ NM(*μ*, *r*, *p*_1_, *p*_2_, … *p*_*n*_), where ***X*** is the random count vector with *n* random components, and *μ*, *r*, *p*_1_, *p*_2_, … *p*_*n*_ are the parameters.

The distribution can be defined in terms of a simple hierarchical model:

1. the random variable X_+_ = ∑_i = 1_^n^X_1_ follows a negative binomial distribution: *X*_+_ ∼ NB(*μ*, *r*),
2. the counts *X*_1_, *X*_2_, … *X*_*n*_, given that *X*_+_ equals *x*_+_, follow a multinomial distribution: ***X*** |{*X*_+_ = *x*_+_} ∼ Multinom(*x*_+_, *p*_1_, *p*_2_, … *p*_*n*_),
3. as a consequence, the probability of observing the count vector ***x*** = (*x*_1_, *x*_2_, …, *x*_*n*_) is:

$$ \begin{array}{l} Prob\left\{\boldsymbol{X}=\boldsymbol{x}\right\}=\\ {} Prob\left\{{X}_{+}={x}_{+}\right\}\  Prob\left\{\boldsymbol{X} = \boldsymbol{x}\kern0.5em \Big|\ {X}_{+}={x}_{+}\right\} = \\ {}\left\{\frac{\varGamma \left(r + {x}_{+}\right)}{\varGamma (r)\ {x}_{+}!}{\left(\frac{\mu }{r+\mu}\right)}^{x_{+}}{\left(\frac{r}{r+\mu}\right)}^r\right\}\ \left\{{x}_{+}!{\displaystyle \prod_{i=1}^n}{\frac{p_i}{x_i!}}^{x_i}\right\}.\end{array} $$

In one version of the algorithm (the ‘independent’ mode), for each hidden state there are *n* + 1 free parameters that characterize a negative multinomial distribution. In another variant (the ‘dependent’ mode, which was used in our analyses), there is only one *r* parameter for all distributions. This reduces the total number of emission parameters from *k* (*n* + 1) to *kn* + 1 (just one more than in ChromHMM) and excludes unrealistic models where different states have wildly different dispersion parameters. The update rules for the parameters of the distribution in the Baum-Welch algorithm can be found in Additional file 1: Section 3.

### Implementation

EpiCSeg has been implemented as an R packages with two main design goals in mind: ease of use and efficiency. The interface is simple and familiar to the large bioinformatics and statistics community using the R language. A command-line interface is also available, for those users not familiar with R. At the same time, most of the time-consuming operations have been developed in C++, parallelized with OpenMP [20] and interfaced with R using the Rcpp package [21], which ensures efficiency and scalability with the number of cores in shared-memory architectures. Beside the functionalities shown in these analyses, such as producing the genome-wide segmentation statistics, EpiCSeg can also be used to learn the same set of states across different datasets and to aggregate replicate ChIP-seq tracks into one. EpiCSeg is available under the GPLv3 license at the website http://github.com/lamortenera/epicseg.

### The supervised annotation

The supervised annotation was made using the gene annotation from GENCODE [16] and cell-type specific RNA-seq and DNase I HS experiments following these simplified steps (for the complete description see Additional file 1: Section 4):

1. We considered only those 200 base pairs bins that do not overlap any assembly gap.
2. For each bin, we counted the number of DNase I tags and the average coverage of RNA-seq read pairs.
3. Based on the quantiles of the DNase and RNA counts per bin, we defined a stringent and a permissive threshold for RNA and a stringent threshold for DNase read counts.
4. Bins with a count larger than the stringent DNAse threshold were classified as DNase bins.
5. DNase bins were further split into DNase+TSS and DNase-TSS bins (respectively, promoter and enhancer environments), depending on the distance from an annotated TSS.
6. Bins with a count larger than the stringent threshold for RNA, smaller than the permissive threshold for DNase, and far from the boundaries where these two conditions start to hold, were classified as RNA bins (transcription environment).
7. Bins that were not classified as RNA or DNase bins and very far away from the boundaries where these two conditions start to hold, were classified as intergenic bins.

### The robustness score

To measure how similar two segmentations are to each other we used the average Jaccard index. The Jaccard index between two sets *A* and *B* is the ratio between the size of the intersection and the size of the union. If |*A*| denotes the size of set *A* (in our setting, a number of base pairs), the Jaccard index can be expressed by the formula $\frac{\left|A\ {\displaystyle \cap }B\right|}{\left|A\ {\displaystyle \cup }B\right|}$. Given two segmentations, and assuming that there is a one-to-one correspondence between states, the average Jaccard index is computed as follows:

1. for each state *s* consider the two sets of base pairs *I*_1_ and *I*_2_, defined as the bins where state *s* occurs in segmentation 1 and 2 respectively
2. measure the Jaccard index *J*_*s*_ between the two sets, defined as ${\mathrm{J}}_{\mathrm{s}} = \frac{\left|{\mathrm{I}}_1{\displaystyle \cap}\;{\mathrm{I}}_2\right|}{\left|{\mathrm{I}}_1{\displaystyle \cup }{\mathrm{I}}_2\right|}$
3. as the final score, consider the average Jaccard index *J* across all states $\mathrm{J} = {\displaystyle {\sum}_{\mathrm{s}\in \mathrm{S}}\frac{\mathrm{Js}}{\left|\mathrm{S}\right|}}$, where |*S*| denotes the number of states.

The one-to-one correspondence between states is chosen as the one that maximizes the average Jaccard index.

## Additional file

Additional file 1. **The Supplementary Material is provided as a .pdf file and it contains additional figures and more detailed information about the computational methods and the results.**

## Abbreviations

bp(s): base pair(s)
ChIP-seq: Chromatin Immunoprecipitation followed by Sequencing
PCR: Polymerase Chain Reaction
TES: Transcription End Site
TSS: Transcription Start Site
