# Supplementary material for: Chromatin segmentation based on a probabilistic model for read counts explains a large portion of the epigenome
Source: Genome Biol. 2015 Jul 24;16(1):151. doi: 10.1186/s13059-015-0708-z (PMC4514447; doi:10.1186/s13059-015-0708-z)
Supplement: Supplementary file 1 — The Supplementary Material is provided as a .pdf file and it contains additional figures and more detailed information about the computational methods and the results. [file 13059_2015_708_MOESM1_ESM.pdf]

Chromatin segmentation based on probabilistic model for reads counts explains a large portion of the epigenome

Supplementary Material

Alessandro Mammana
Ho-Ryun Chung

Contents

1 Notation **1**

2 The Initialization Algorithm **2**

3 The probabilistic model of EpiCSeg **3**
3.1 Model definition . . . 3
3.2 Parameter estimation through Expectation Maximization . . . 4
3.3 The Baum-Welch algorithm . . . 4
3.4 Update rules for the Negative Multinomial distribution . . . 5
3.5 Computational considerations . . . 6

4 Supervised annotation **6**
4.1 RNA-seq processing . . . 6
4.2 DNase-I HS processing . . . 7
4.3 Annotation criteria . . . 7

5 Segmentation statistics and plots **9**
5.1 Additional performance scores . . . 9
5.2 Scores dependence on the number of states . . . 11
5.3 Comparison with Segway . . . 12
5.4 Replicate similarity . . . 16
5.5 Error rates vs read coverage . . . 17
5.6 EpiCSeg's model robustness to shifts of the binning offset . . . 18
5.7 Genomic distribution of the chromatin states . . . 20

1 Notation

This section introduces the notation used in Section 2 and 3. We will assume that there are g genomic regions to be analyzed, that a region i is composed of L_i bins, and that we are analyzing n different histone marks. The input data for a region i consists of a count matrix $C^{(i)}$ with L_i

rows and n columns containing the number of reads from each mark mapping to each bin. In the HMM framework, the i-th sequence of observations is encoded in the count matrix $C^{(i)}$, and the b-th observation symbol in the i-th sequence is the b-th row in $C^{(i)}$, composed of n counts.

2 The Initialization Algorithm

The Baum-Welch algorithm used by EpiCSeg for fitting the Hidden Markov Model needs an initial value for all the model parameters. Those parameters will eventually be fit to the data, but the possible final values depend on the initial ones, which makes this choice important. The goal of the initialization procedure is to determine a good starting point for all parameters of the Hidden Markov Model. The initialization algorithm developed for EpiCSeg is based on the following ideas:

- Initializing the parameters for k chromatin states can be done by clustering the observation vectors into k clusters and therefore disregarding the order of the observations.
- Many small clusters (seeds) can be merged and reduced to k clusters using hierarchical clustering.
- Principal Component Analysis (PCA) can be applied on the count matrix to find good seeds.

Let k be the desired number of clusters and C the matrix with n columns and $L = \sum_{i=1}^{g} L_i$ rows obtained by combining all matrices $C^{(i)}$. C and k are the input of the algorithm while the output are k sets of bins. These sets are not exactly a clustering, because they can overlap, and not even proper sets, as the same bin in the same set can appear more than once. However it is easy to fit the probabilistic model starting from them (not discussed).

The algorithm performs the following steps:

1. PCA is performed on the count matrix C. This results in a coordinate matrix P with the same dimensions as C but where the columns correspond to principal components (PCs).
2. For each PC, *nlev* seeds are computed. Here seed means a subset of the bins and *nlev* is a parameter of the initialization algorithm. The seeds deriving from a PC p result from partitioning the rows of matrix P into *nlev* groups of equal size according to the intensity of the p-th column. So, for instance, the first group is formed by the $L/nlev$ rows with the lowest values in column p. This yields a total of $nlev \cdot k$ seeds.
3. For each seed the parameters of a negative multinomial distribution $NM(\mu, r, p_1, p_2, ...p_n)$ are determined. The r parameter is fitted for all seeds according to some properties of matrix C (not discussed), while the other parameters are estimated by maximum likelihood on the data points identified by the seed.
4. A distance matrix between seeds is computed. The distance between two seeds is defined as the symmetrized Kullback-Leibler divergence between the two corresponding negative multinomial distributions.
5. The distance matrix is used as input to the hierarchical clustering algorithm with average linkage. The *hclust* function available in *R* is used. Hierarchical clustering produces a tree where the leaves represent the different seeds, internal nodes represent clusters of seeds and where the length of a branch represents the similarity between two nodes.

6. The tree produced by the hierarchical clustering algorithm is cut at a distance from the root such that the resulting tree has exactly k nodes. Each node is a group of seeds that will be used to initialize a chromatin state.

3 The probabilistic model of EpiCSeg

Hidden Markov Models (HMMs) are very well known in literature and many of the equations presented here can be found in textbooks covering the topic, such as [1]. A brief introduction, however, is necessary to present the update equations for the negative multinomial distributions, which represent a novel contribution of this work.

3.1 Model definition

For ease of discussion we will assume that there is only one count matrix C corresponding to one genomic region with a total of L bins. The t-th row of the count matrix, where $1 \leq t \leq L$, will be denoted as $\mathbf{c}_t$, the count in the t-th bin corresponding to the m-th mark, where $1 \leq m \leq n$, will be denoted as c_{tm} and the sum of the counts in the t-th row will be denoted as c_{t+}, *i.e.* $c_{t+} = \sum_{m=1}^{n} c_{tm}$.

The main assumption of the model is that there are k hidden states and that the sequence of L observations $\mathbf{c}_t$ is explained by a hidden sequence of L states. The random variable X_t denotes the hidden state at position t and the random variable Y_t represents the observation at time t (in this context Y_t is always known and equals $\mathbf{c}_t$, but the random variable notation is useful to explain the probabilistic model).

The complete set of model parameters, denoted as θ, consists of the parameters $\boldsymbol{\pi}, A, M_1, M_2, ...M_k$, where:

- $\boldsymbol{\pi}$, the *initial probabilities*, are a vector of k probabilities summing up to one where π_s specifies $Prob\{X_1 = s|\theta\}$.
- A, the *transition probabilities*, are a square matrix of size k, where the element in row u and column v, denoted as a_{uv}, specifies $Prob\{X_{t+1} = v|X_t = u, \theta\}$, independently of the position t in the sequence. In A each row u sums up to one, *i.e.* $\sum_{v=1}^{k} a_{uv} = 1$.
- M_s is the parameter set that determines the *emission probabilities* relative to state s, which follows a Negative Multinomial distribution with parameters $\mu_s, r_s, p_{s1}, p_{s2}, ...p_{sn}$. The Negative Multinomial distribution can be seen as a combination between the Negative Binomial distribution, specified by parameters μ_s and r_s, and the Multinomial distribution, specified by the parameters $p_{s1}, p_{s2}, ...p_{sn}$. In formulas

$$Prob\{Y_t = \mathbf{c}_t|X_t = s, \theta\} = f_{NM}(\mathbf{c}_t; M_s) = f_{NB}(c_{t+}; \mu_s, r_s) \cdot f_{Multinom}(\mathbf{c}_t; p_{s1}, p_{s2}, ...p_{sn}), \quad (1)$$

where

$$f_{NB}(c_{t+}; \mu_s, r_s) = \frac{\Gamma(r_s + c_{t+})}{\Gamma(r_s)(c_{t+})!} \left(\frac{\mu_s}{\mu_s + r_s}\right)^{c_{t+}} \left(\frac{r_s}{\mu_s + r_s}\right)^{r_s},$$

and

$$f_{Multinom}(\mathbf{c}_t; p_{s1}, p_{s2}, ...p_{sn}) = (c_{t+})! \prod_{m=1}^{n} \frac{p_{sm}^{c_{tm}}}{c_{tm}!}.$$

In an important variant of the EpiCSeg model all r_s variables are constrained to have the same value. This strategy has proved effective in avoiding overfitting and excluding some unrealistic models where different states s have wildly different values of the parameter r_s. This variant of the model is called the *dependent mode*, while the variant where the r_s parameters are independent is called the *independent mode*. Unless otherwise specified this discussion focuses on the independent mode.

Given this model, the probability that the sequence of observations $\mathbf{Y} = Y_1, Y_2, ...Y_L$ equals the count matrix (*i.e.*, that $Y_t = \mathbf{c}_t$ for $1 \leq t \leq L$), and that the sequence of hidden states $\mathbf{X} = X_1, X_2, ...X_L$ equals a certain sequence $\mathbf{s} = s_1, s_2, ..s_L$, with $\mathbf{s} \in \{1, 2, ...k\}^L$, is given by

$$Prob\{\mathbf{X} = \mathbf{s}, \mathbf{Y} = C|\theta\} = \pi_{s_1} f_{NM}(\mathbf{c}_1; M_{s_1}) \prod_{t=2}^{L} a_{s_{t-1}s_t} f_{NM}(\mathbf{c}_t; M_{s_t}),$$

and the overall probability of the observed data can be obtained by summing the contributions of all possible paths:

$$Prob\{\mathbf{Y} = C|\theta\} = \sum_{\mathbf{s} \in \{1,2,...k\}^L} Prob\{\mathbf{X} = \mathbf{s}, \mathbf{Y} = C|\theta\}.$$

3.2 Parameter estimation through Expectation Maximization

To estimate the parameters of the model we follow a Maximum Likelihood approach, *i.e.* we seek to estimate the set of parameters $\theta^{(opt)}$ that maximizes the overall probability of the observed data

$$\theta^{(opt)} = \arg\max_{\theta} Prob\{\mathbf{Y} = C|\theta\}.$$

Unfortunately this is a very difficult problem which typically needs to be dealt with with some iterative procedure, such as Expectation Maximization (EM). With Expectation Maximization, starting from a parameter set $\theta^{(1)}$, a sequence of parameter sets $\theta^{(2)}, \theta^{(3)}, \theta^{(4)}...$ is computed using the following update rule:

$$\theta^{(i+1)} = \arg\max_{\theta} \sum_{\mathbf{s} \in \{1,2,...k\}^L} Prob\{\mathbf{X} = \mathbf{s}|\mathbf{Y} = C, \theta^{(i)}\} \log\{Prob\{\mathbf{X} = \mathbf{s}, \mathbf{Y} = C|\theta\}\} \tag{2}$$

It can be shown that using this strategy $Prob\{\mathbf{Y} = C|\theta^i\}$ is always less or equal to $Prob\{\mathbf{Y} = C|\theta^{(i+1)}\}$ (see, for instance, [3]). The sequence typically stops when there is no further increase in the objective function and the algorithm is said to converge. The major drawbacks of this approach are that the final parameters of the sequence are not guaranteed to be a global optimum and different initial parameters can lead to different final parameters. The Expectation Maximization strategy applied to Hidden Markov Models is known as the Baum-Welch algorithm.

3.3 The Baum-Welch algorithm

The Baum-Welch algorithm uses the *posterior probabilities* $\gamma_s(t)$ and the quantities $\xi_{uv}(t)$. They depend on the last set of parameters $\theta^{(i)}$ and they are defined in the following way:

- $\gamma_s(t) = Prob\{X_t = s|\mathbf{Y} = C, \theta^{(i)}\}$
- $\xi_{uv}(t) = Prob\{X_{t-1} = u, X_t = v|\mathbf{Y} = C, \theta^{(i)}\}$

Those quantities, which are computed using the Forward-Backward strategy (see [1]), allow to separate the maximization problem in Equation 2 into three independent and simpler maximization problems:

$$\theta^{(i+1)} = \arg\max_{\theta} \left\{ \sum_{s=1}^{k} \gamma_s(1) \log \pi_s + \sum_{t=2}^{L} \sum_{u=1}^{k} \sum_{v=1}^{k} \xi_{uv}(t) \log a_{uv} + \sum_{t=1}^{L} \sum_{s=1}^{k} \gamma_s(t) \log f_{NM}(\mathbf{c}_t; M_s) \right\} \quad (3)$$

Solving the maximization problem in Equation 3 separately for each parameter yields the following well known update rules for the initial and transition probabilities:

- $\pi_s^{(i+1)} = \gamma_s(1)$
- $a_{us}^{(i+1)} = \frac{\sum_{t=2}^{L} \xi_{us}(t)}{\sum_{v=1}^{k} \sum_{t=2}^{L} \xi_{uv}(t)}$

3.4 Update rules for the Negative Multinomial distribution

The factorization shown in Equation 1 allows to further split the maximization problem into

- $\mathbf{p}_s^{(i+1)} = \arg\max_{\mathbf{p}_s} \sum_{t=1}^{L} \gamma_s(t) \log f_{Multinom}(\mathbf{c}_t; \mathbf{p}_s)$,
- $\mu_s^{(i+1)}, r_s^{(i+1)} = \arg\max_{\mu_s, r_s} \sum_{t=1}^{L} \gamma_s(t) \log f_{NB}(c_{t+}; \mu_s, r_s)$.

This yields:

- $p_{sm}^{(i+1)} = \frac{\sum_{t=1}^{L} \gamma_s(t) c_{tm}}{\sum_{t=1}^{L} \gamma_s(t) c_{t+}}$,
- $\mu_s^{(i+1)} = \frac{\sum_{t=1}^{L} \gamma_s(t) c_{t+}}{\sum_{t=1}^{L} \gamma_s(t)}$,

and for the r_s parameters:

$$r_s^{(i+1)} = \arg\max_{r_s} \sum_{t=1}^{L} \gamma_s(t) \log f_{NB}(c_{t+}; \mu_s^{(i+1)}, r_s). \quad (4)$$

Unfortunately there is no closed formula for the last maximization problem, which needs to be solved numerically as a one dimensional optimization problem. It is known, however, that there exist only one local maximum, which is also a global maximum [2]. In EpiCSeg a variant of the Brent algorithm is used for this task.

In the dependent mode of the EpiCSeg model, *i.e.* when all parameters r_s are constrained to have the same value r, all the above update equations remain valid except Equation 4, which becomes

$$r^{(i+1)} = \arg\max_{r} \sum_{s=1}^{k} \sum_{t=1}^{L} \gamma_s(t) \log f_{NB}(c_{t+}; \mu_s^{(i+1)}, r), \quad (5)$$

and can be solved numerically as in the previous case.

3.5 Computational considerations

The update formulas 4 and 5 can be very costly to compute. The optimization function consists of a summation over all the L bins of a quantity that depends on the f_{NB} function, which is a very costly function. Numerical methods need to evaluate the optimization function, or its derivative, a number N_r of times before returning the updated value for r_s, where typical values for N_r range from 10 to 30 iterations, and where these evaluations need to be done serially, *i.e.* they cannot be executed in parallel. Assuming that $L \sim 1.5 \cdot 10^7$ (as for a whole human genome and with a bin size of 200 bps) and $N_t \sim 20$, the Expectation Maximization algorithm would need to evaluate the f_{NB} function about $3 \cdot 10^8$ times at each iteration.

This problem can be alleviated by grouping together evaluations of the $f_{NB}(c_{t+}; \mu_s^{(i+1)}, r_s)$ function with the same c_{t+} value. Let

- $D = \{c_{t+} : 1 \leq t \leq L\}$, and
- $\delta_s(d) = \sum_{t=1}^{L} \gamma_s(t)[c_{t+} = d]$,

where the expression delimited by square brackets evaluates to one when the expression inside it is true and to zero otherwise. Then the update formulas 4 and 5 can be rewritten respectively as

$$r_s^{(i+1)} = \arg\max_{r_s} \sum_{d \in D} \delta_s(t) \log f_{NB}(d; \mu_s^{(i+1)}, r_s),$$

and

$$r^{(i+1)} = \arg\max_{r} \sum_{d \in D} \sum_{t=1}^{L} \delta_s(t) \log f_{NB}(d; \mu_s^{(i+1)}, r),$$

respectively. Considering that D, even in genome-wide datasets, rarely contains more than 10^4 elements, the f_{NB} function now needs to be evaluated $1.5 \cdot 10^3$ times less frequently.

4 Supervised annotation

The aim of the supervised annotation is to provide a benchmark dataset to evaluate the efficiency of a segmentation algorithm. This section describes the procedure used to derive such an annotation.

The input to the procedure is a RNA-seq and a DNase-I hypersensitivity experiment from the same cell type as the histone mark experiments used for the segmentation. Additionally the procedure uses the annotated transcripts available from the GENCODE database, version 19 (Ensembl 74). More precisely, all annotations of type "transcript" and of level 1 and 2 where considered. The binning scheme used for this procedure is the same as the one used for segmentation, *i.e.* the bin have size 200 base pairs, with the difference that all bins overlapping a non-assembled region of the genome were discarded.

4.1 RNA-seq processing

In the RNA-seq tracks, which are paired-end sequencing experiments, regions ranging from the leftmost to the rightmost coordinate of a mapped read pair were treated as unstranded transcribed regions. The coverage per base pair was computed as the number of such transcribed regions that overlap the base pair, and the transcription signal per bin was defined as the average coverage in the bin. The top 15% bins were considered transcribed.

4.2 DNase-I HS processing

In the DNase-I hypersensitivity tracks, which are single-end sequencing experiments, the accessibility signal per bin was defined as the number of reads with a 5' end mapping within the bin. The top 2% bins were considered accessible and the top 0.8% were considered strongly accessible.

4.3 Annotation criteria

The following criteria were used to define each chromatin environment:

- A **RNA** bin was defined as a transcribed and non-accessible bin and such that all the 10 bins to the right and to the left are also transcribed and non-accessible.
- A **DNase** bin was defined as a strongly accessible bin. If the bin is closer than 500 base pairs to an annotated TSS it was considered a **DNase+TSS** bin, otherwise a **DNase-TSS** bin.
- A bin was annotated as **intergenic** if that bin, as well as the 100 bins to the left and to the right, are neither a DNase nor a RNA bin.

Figure 1 shows the average distribution of the chromatin environments with respect to the annotated genes.

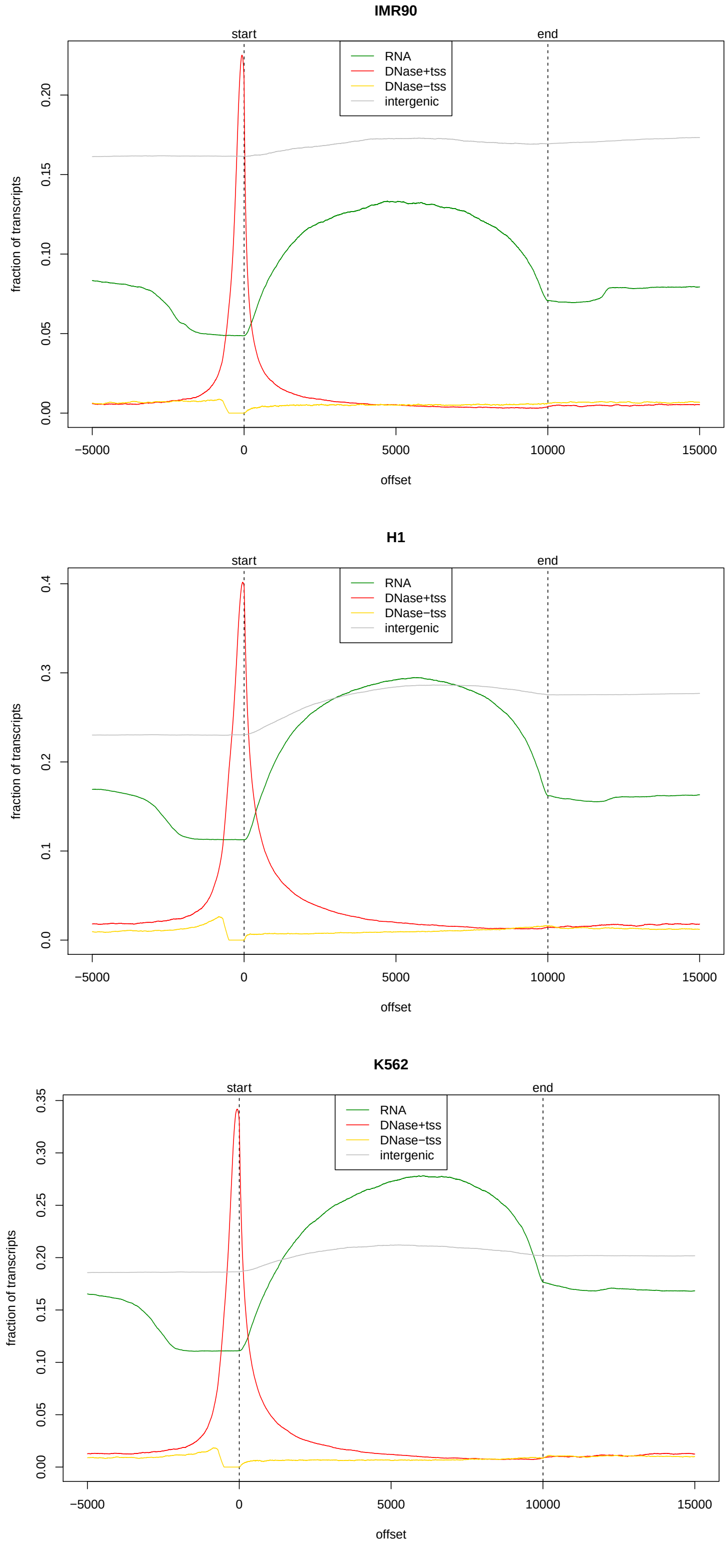

Figure 1: Distribution of the chromatin environments with respect to annotated genes. From top to bottom the figure shows data for the `IMR90`, `H1` and `K562` datasets. Each plot shows the fraction of annotated transcripts having a bin of a certain type at a certain position of the transcript. Positions from the TSS and the TES have been rescaled to fit a common length.

5 Segmentation statistics and plots

5.1 Additional performance scores

We measured the association between the supervised annotation and a segmentation in two different ways. All of them are based on a contingency table where the rows represent chromatin environments, the columns chromatin states, and each cell contains the number of bins annotated with a certain chromatin environment and state. Let the index E denote the set of possible chromatin environments and S the possible chromatin states. Moreover, let N_+ denote the total number of bins and $N(e, s)$ the number of bins that have the environment label e and state label s. From these counts we can derive the following proportions:

$$P_{ES}(e, s) = N(e, s)/N_+; P_E(e) = \sum_{s \in S} P_{ES}(e, s); P_S(s) = \sum_{e \in E} P_{ES}(e, s).$$

The **mutual information** measures the association between the two annotations:

$$MI = \sum_{e \in E, s \in S} P_{ES}(e, s) \log P_{ES}(e, s)/(P_E(e)P_S(s)).$$

However, we needed to correct for the fact that the distribution of the chromatin environments is highly imbalanced. Some environments, such as the intergenic environment, occur much more often and influence the performance score much more that others, such as the DNase+TSS environment. This leads to large oscillations of the score with small perturbations of the input data and to the paradox that the most biologically interesting states, such as those related to the DNase+TSS environment, play a lesser role in the performance score. To correct for that we use proportions $\tilde{P}$ normalized for the chromatin environment frequency: $\tilde{P}_{ES}(e, s) = P_{ES}(e, s)/\{P_E(s)|E|\}$, and $\tilde{P}_E, \tilde{P}_S$ are defined starting from $\tilde{P}_{ES}$ as above. The (corrected) mutual information (see Figure 2) is defined as above, but using the corrected frequencies:

$$MI(\text{corrected}) = \sum_{e \in E, s \in S} \tilde{P}_{ES}(e, s) \log \tilde{P}_{ES}(e, s)/(\tilde{P}_E(e)\tilde{P}_S(s)).$$

Note that if (X_E, X_S) are two random variables with joint probability distribution given by $\tilde{P}_{ES}$, the above formula can be related to the conditional entropy of X_E given X_S:

$$MI(\text{corrected}) = |E| \log |E| - H(X_E|X_S).$$

Lastly, we measured how similar each pair of chromatin environments is in terms of chromatin states frequency (see Figure 3) using the following measure, that we called **mutual similarity**:

$$MS = \sum_{e_1 \in E} \sum_{e_2 \in E \setminus \{e_1\}} \sum_{s \in S} \tilde{P}_{ES}(e_1, s)\tilde{P}_{ES}(e_2, s).$$

The **runtime** of the two algorithms was measured by running a whole-genome segmentation with 10 states on each dataset. Each genome-wide dataset consists of a matrix with 15181508 bins (rows) and with 27, 26, 12 and 12 marks (columns) for the `IMR90`, `H1`, `K562_1` and `K562_2` datasets respectively. We used the default parameters for each algorithm except the number of cores, which was set to 10. The computer used for the comparison is a 64x AMD Opteron 6282 SE with 517

GB or RAM (even though each algorithm needed at most 2 GB). The result of the measurements can be seen in Figure 4, and it suggests that both ChromHMM and EpiCSeg are fast enough for our needs.

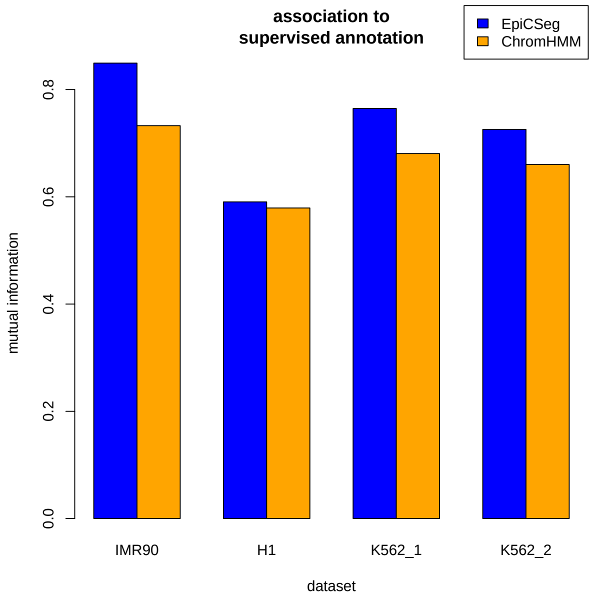

Figure 2: Mutual information between the supervised annotation and the unsupervised segmentations. The higher the balanced mutual information, the better the score.

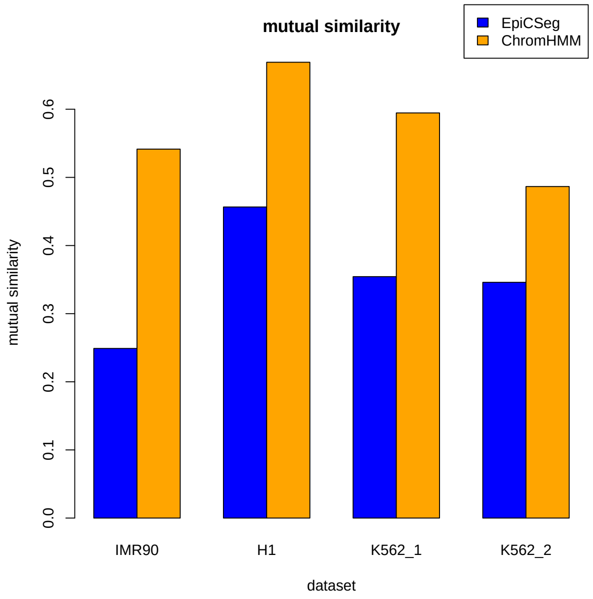

Figure 3: Mutual similarity between states. The lower the similarity, the better the score.

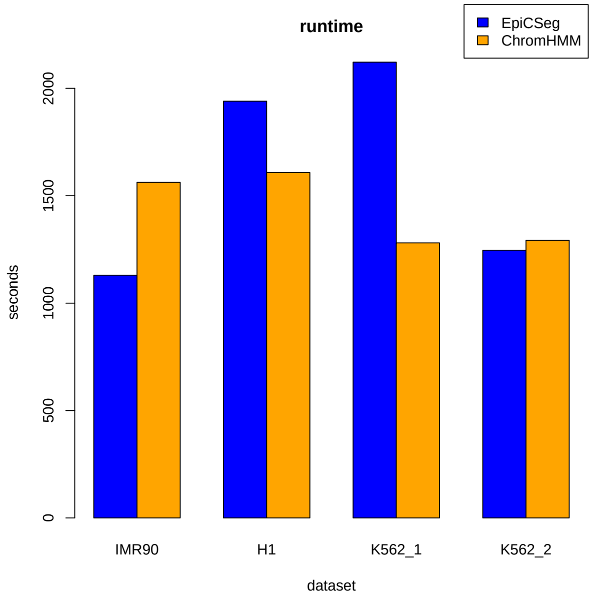

Figure 4: Number of seconds required for a whole genome segmentation.

5.2 Scores dependence on the number of states

We measured the scores reported in the main document varying the number of states from 2 to 40. See the main document for a description of each score. For practical reasons we limited the input data to the first chromosome only (chr1). The low R^2 with the `IMR90` dataset is most likely due to a much smaller sequencing coverage of the RNA-seq experiment (roughly 10 times less reads than in the other datasets).

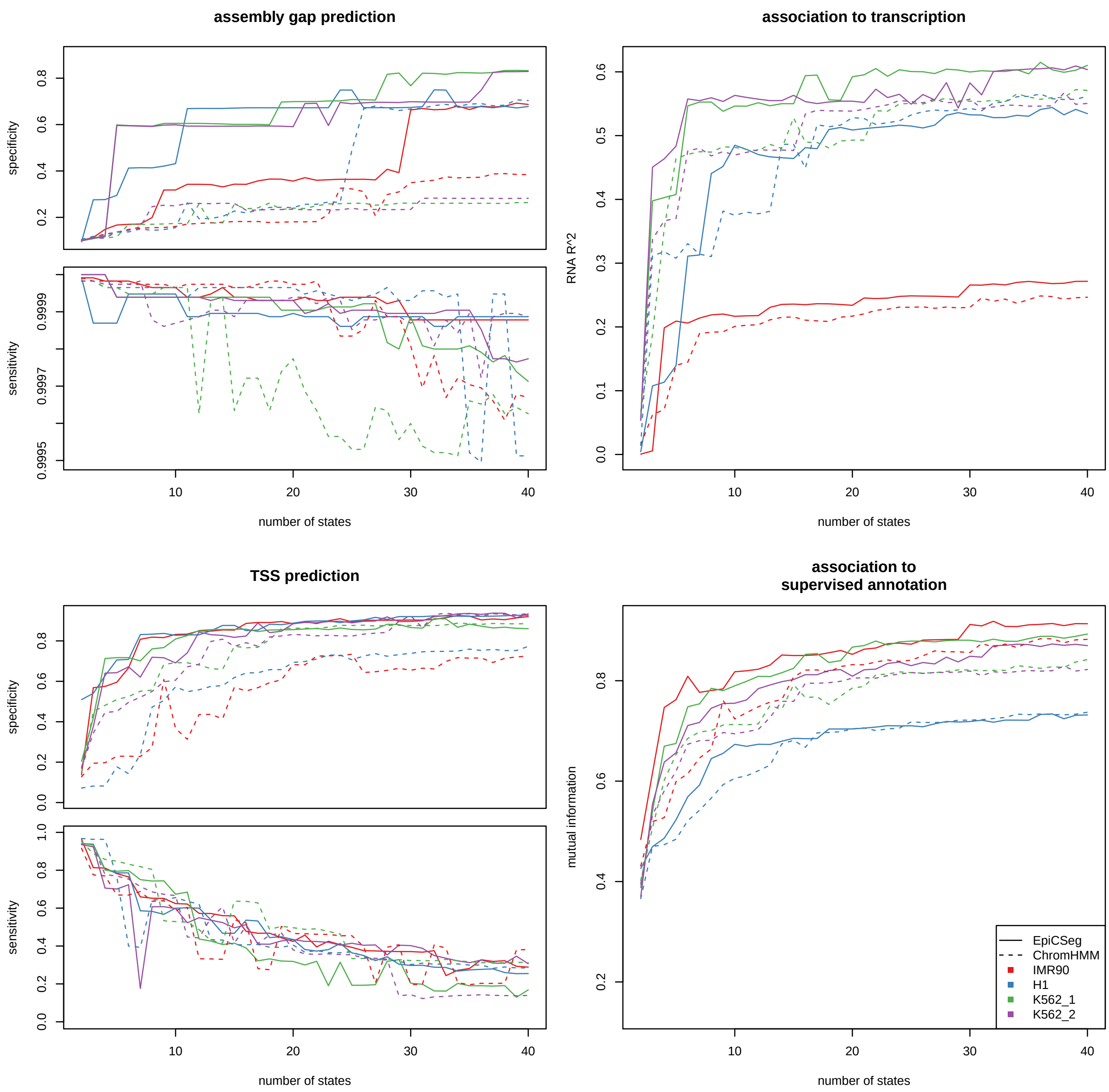

Figure 5: Performance scores described in the main document as a function of the number of states.

5.3 Comparison with Segway

The comparison with Segway poses a challenge both because of its larger runtime and because it works at a single base pair resolution. While also EpiCSeg and ChromHMM can run at a single base pair resolution, aggregating counts from adjacent base pairs has important smoothing properties, both for the segmentation algorithms and for our validation procedure. These smoothing properties play an indispensable role in our view of chromatin states. To mention one of these properties,

binning reduces the oscillations in read counts due to nucleosome positioning. Even though in other contexts it might be desirable to discern between linker and nucleosomal DNA, we consider a promoter region, typically characterized by an array of positioned nucleosomes, as a single entity, which should be annotated by a single segment.

In the computation of our performance scores, for Segway, we converted the state assignments to each base into state assignments to each bin. This was done by picking the most abundant state in each bin (draws, which occurred for less than 1% of the bins, were resolved by picking randomly one of the most abundant states). EpiCSeg and ChromHMM, by contrast, were run using the binning scheme. All other settings are as reported in the main document.

We dealt with the runtime problem by running all segmentation algorithms only on chromosome 21. ChromHMM and EpiCSeg perform training and prediction as a single task, while with Segway we first used the 'TRAIN' task, and then the 'IDENTIFY' task. However, Segway runtime was still too large to allow for training on the whole chromosome. We restricted the training task on the region chr21:37313025-38619844 (zero-based, left-inclusive, right-exclusive coordinates), which is about 1.3 megabases large (2.7% of the whole chromosome) and gene-rich (but also with some gene-empty subregions). Even using these settings Segway was about 400 times slower than EpiCSeg and ChromHMM (see Figure 6).

In Figure 7 we report the results of the comparison using the performance scores described in the main document. The results show that Segway's performance is, in general, considerably lower compared to ChromHMM and EpiCSeg. As an exception, Segway achieves a high precision in TSS prediction task, however this is also accompanied by a low sensitivity. From Figure 8 it can be seen how Segway tends to assign most of the base pairs to a few states. It can also be seen that the promoter-associated state in Segway seems to be affected by the nucleosome pattern expected at promoters, which consists of a nucleosome-depleted region right before the TSS and positioned nucleosomes after and before. According to a view of chromatin states others than the one implicitly adopted in this paper, this might be a desirable behaviour.

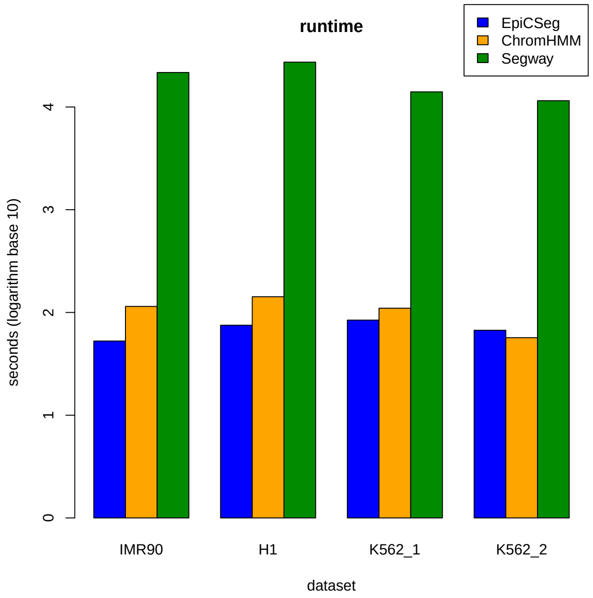

Figure 6: Runtime of the algorithms including Segway (only chromosome 21). Each algorithm was granted 10 cores (even though none of the algorithms made full use of them).

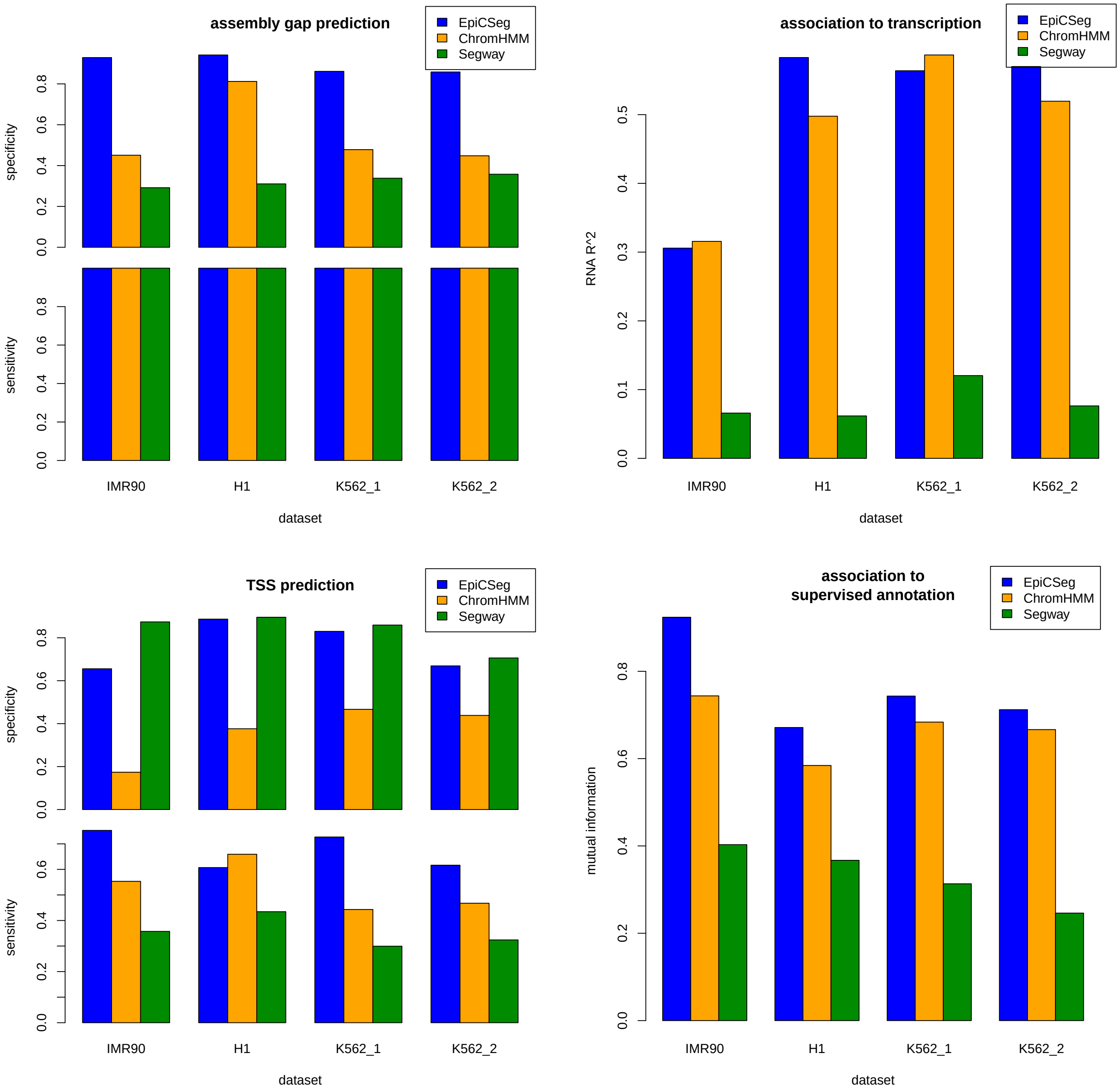

Figure 7: Performance scores described in the main document including Segway (only chromosome 21).

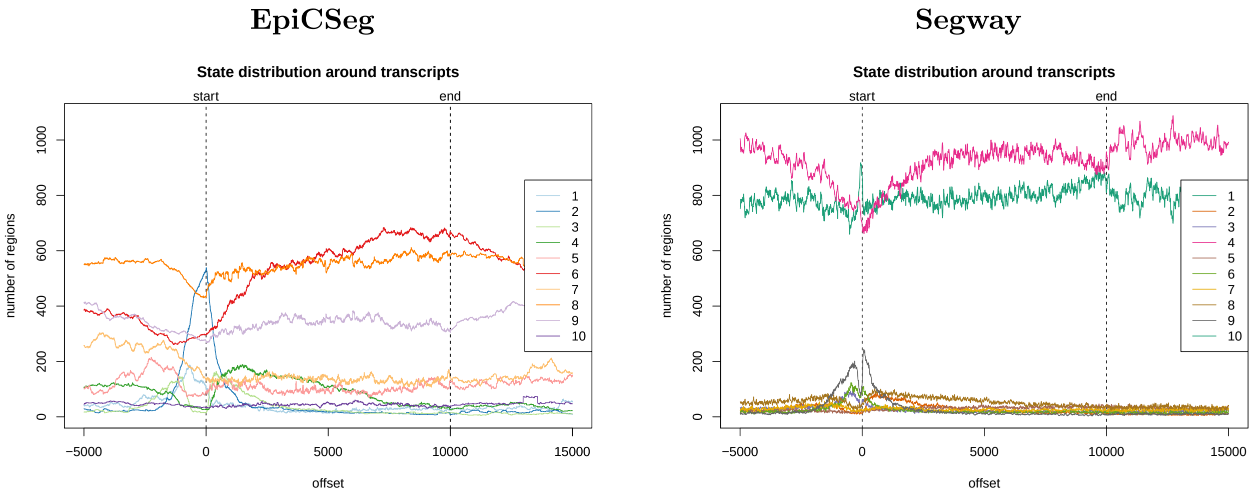

Figure 8: State distribution around transcripts in the `K562_1` datasets using Segway (only chromosome 21). For Segway, the states have been identified at a single base pair resolution, but because the resulting state distribution around transcripts was very noisy, the curves shown above have been smoothed using a running average with a window size of 17 base pairs.

5.4 Replicate similarity

We computed how similar the datasets `K562_1` and `K562_2` are by measuring the Pearson correlation coefficient and the total number of reads (Figure 9) per replicate pair. Note that these statistics rely on the same binning scheme used for the segmentation, *i.e.* each sample is considered as a vector containing the read counts per genomic bin. The first plot shows that the experiments are indeed strongly correlated, suggesting that they are a reflection of the same biological processes. However, there are considerable differences in sequencing coverage.

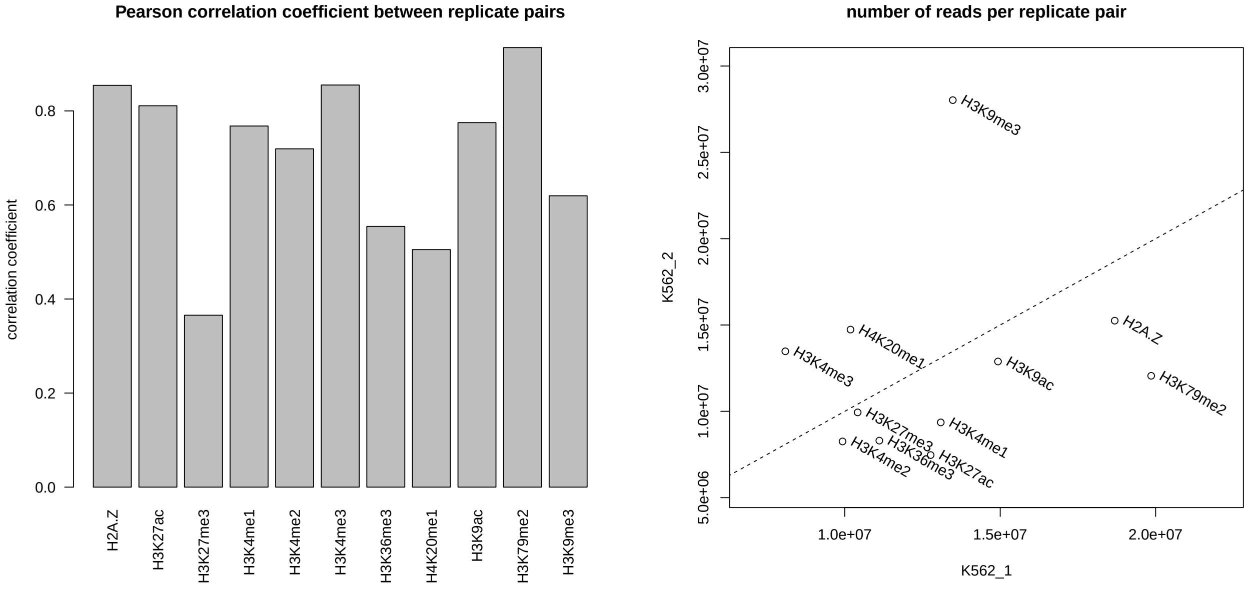

Figure 9: Similarity of each replicate pair. On the left, the Pearson correlation coefficient between replicate pairs. On the right, the total read count for each replicate pair. In both plots each replicate is transformed to a vector containing the read counts per bin.

5.5 Error rates vs read coverage

We studied how the disagreement between segmentations from replicate datasets depends on the total read count per bin. As a result of the robustness analysis presented in the main document, for each algorithm and for each genomic bin in a certain chromosome we have a match or a mismatch depending on whether for that chromosome the segmentations from the `K562_1` and `K562_1` datasets differ in that bin. Next, bins are grouped according to the total read count across marks (computed from the `K562_1` dataset), so that each group consists of at least 500 bins. The mismatch ratio, or error rate, is the ratio of bins in a group where a mismatch occurs. Figure 10 shows how the error rates depend on the read counts.

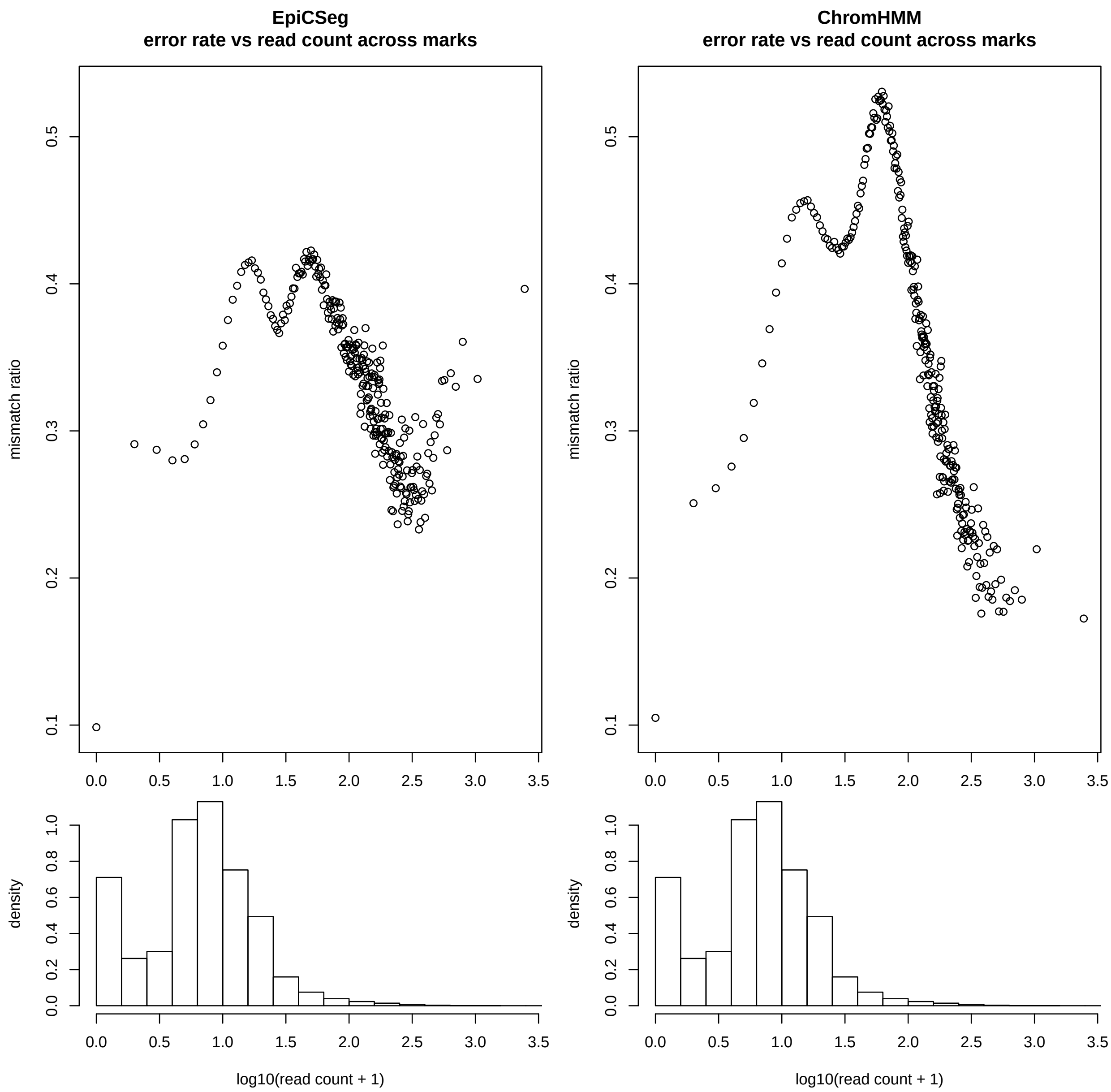

Figure 10: Error rates vs read coverage. The left and right sides relate to EpiCSeg and ChromHMM, respectively. The plots at the top show how the error rates depend on the read counts. Mismatches between bins result from the robustness analysis described in the main document. Mismatch ratios, or error rates, are the ratios of bins in a group where a mismatch occurs. Groups are defined by the total read count across marks in each bin, so that each group consists of at least 500 bins. At the bottom, two identical histograms showing how many bins belong to each group.

5.6 EpiCSeg's model robustness to shifts of the binning offset

The average Jaccard index is an algorithm-independent quantitative measurement of the similarity between two segmentations which is based on finding a correspondence between states. Here,

instead of looking at the segmentations, we look at the model parameters learnt by EpiCSeg and see how different they are. This is shown in figure 11 for all datasets. The figure suggests that corresponding states are, most of the times, indistinguishable, or, when the model converges to a different local minimum, considerably different.

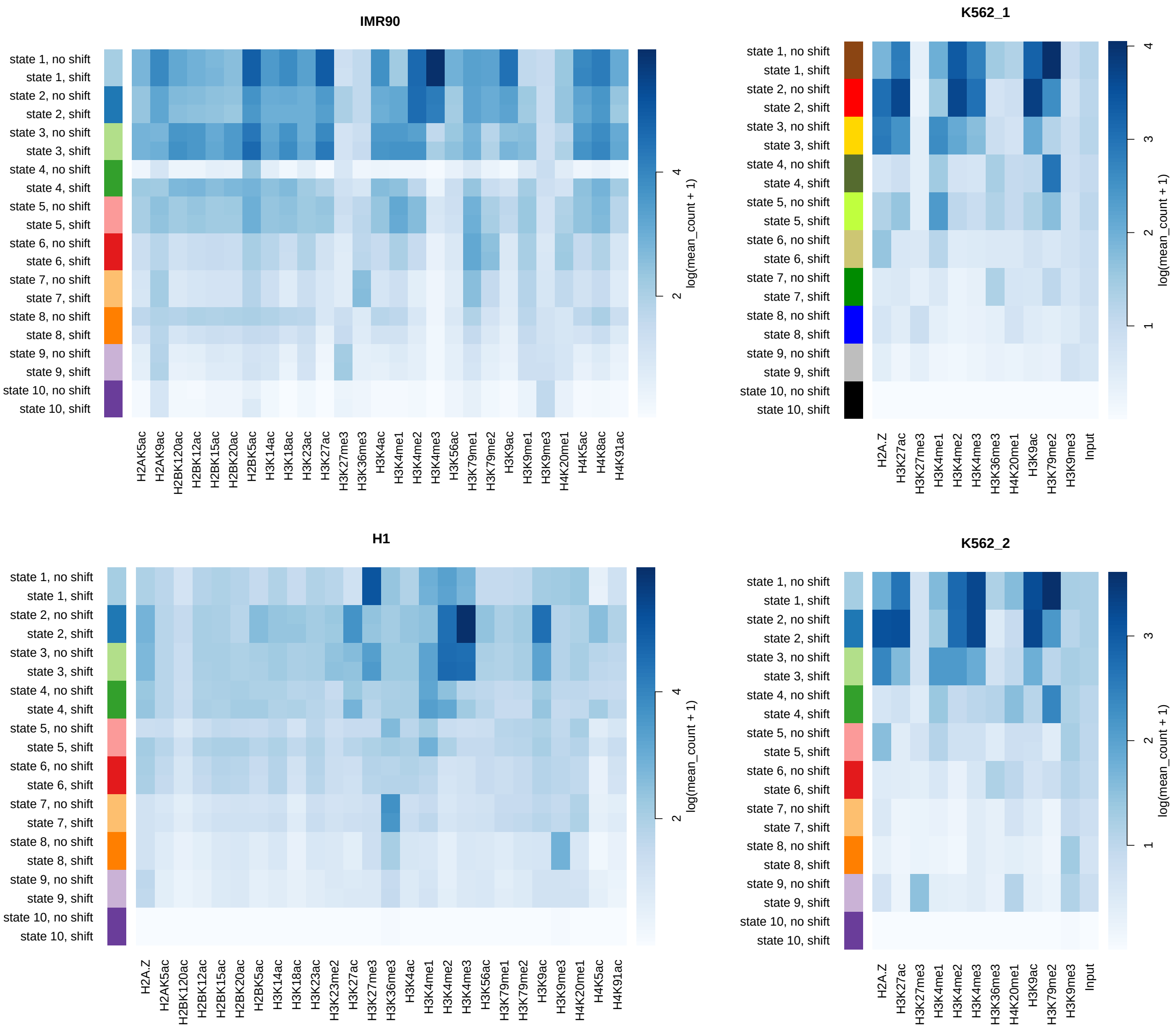

Figure 11: Agreement between models after a shift of the binning scheme. Two models with the same number of states have been trained from the same experiments but counting the reads using a different binnign scheme. In the first binning scheme, labeled as ‘no shift’, the first bin of each chromosome starts at the first base pair, in the second, labeled as ‘shift’, all bins have been shifted by 100 base pair. Next, the two sets of states have been matched so as to maximize the overall agreement of the segmentations, measured with the average Jaccard index. The heatmaps show for each dataset the parameters of the two different model by putting corresponding states next to each other.

5.7 Genomic distribution of the chromatin states

In the Figures 12, 13, 14 and 15 we report the summary statistics described in the main document for all datasets.

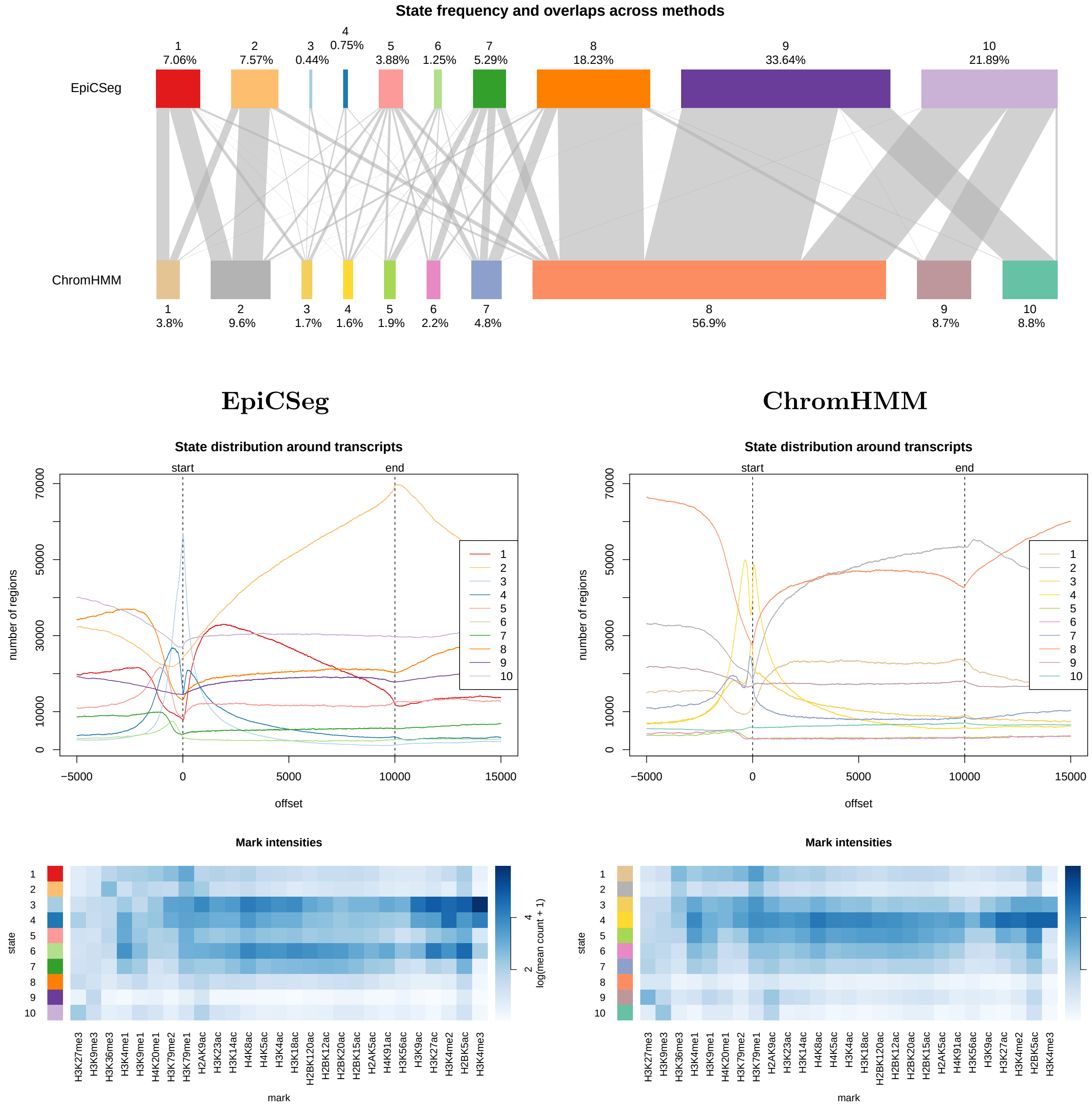

Figure 12: Genomic distribution of chromatin states in the `IMR90` dataset. The choice of the colors is arbitrary.

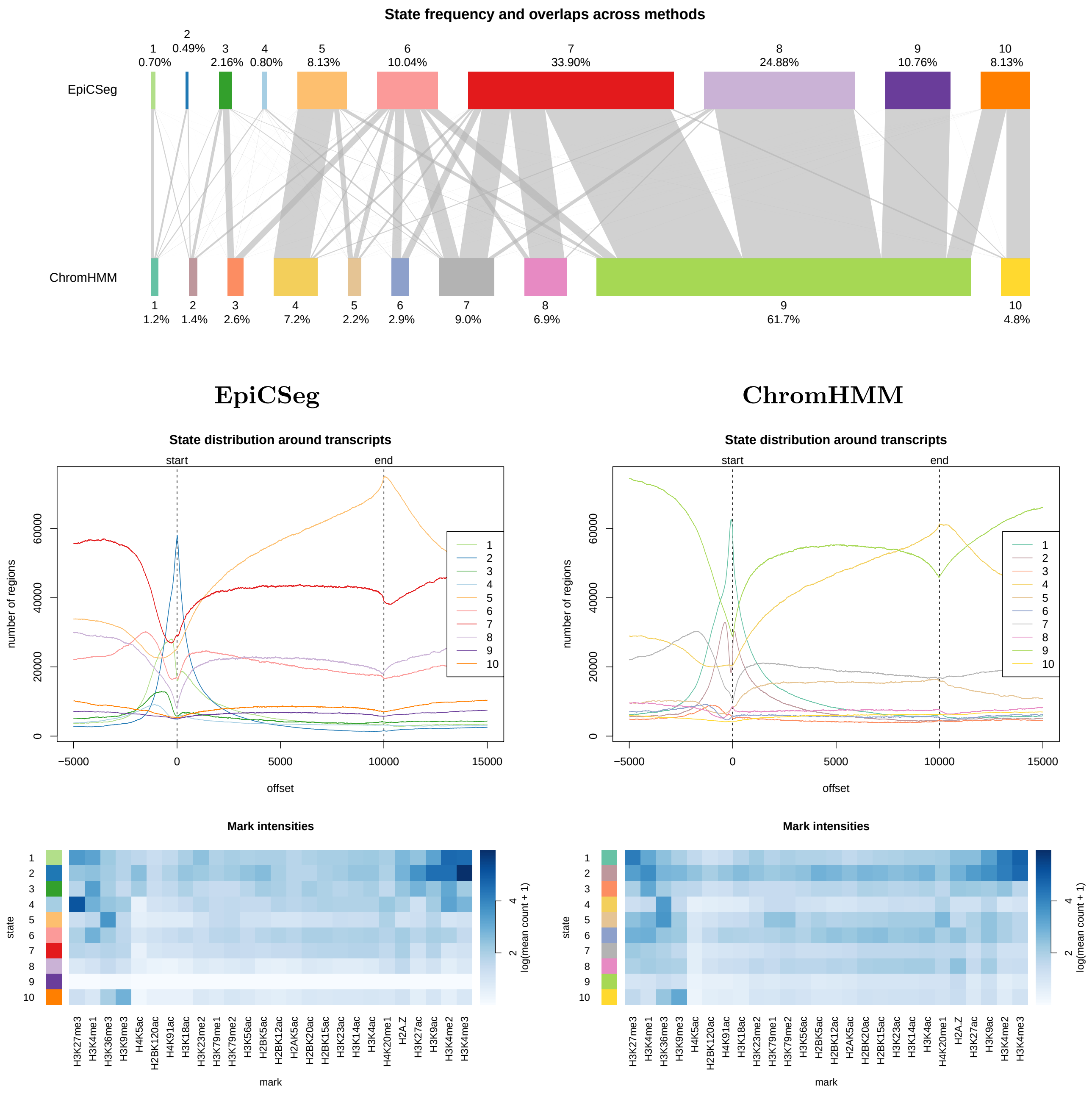

Figure 13: Genomic distribution of chromatin states in the H1 dataset. The choice of the colors is arbitrary.

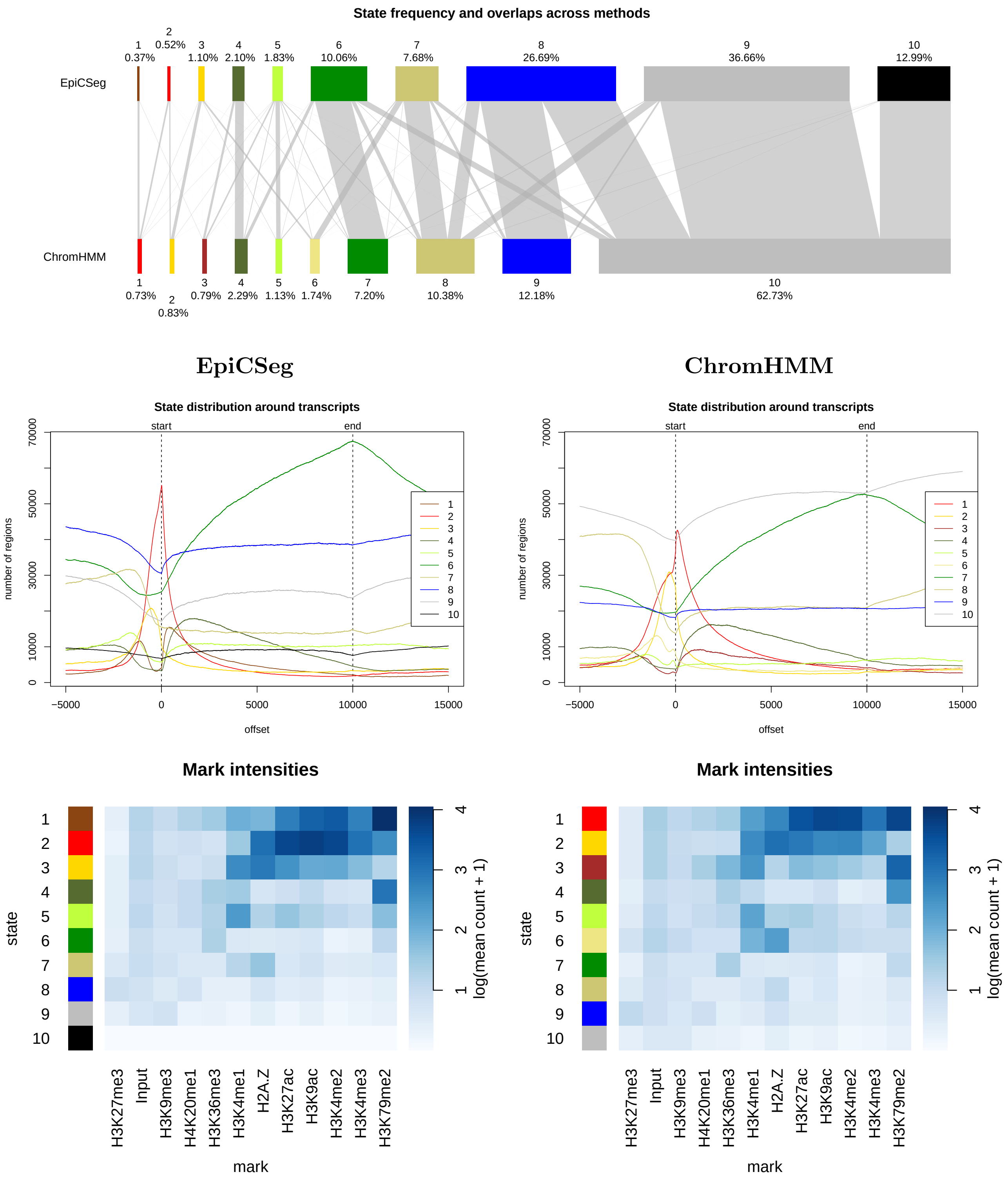

Figure 14: Genomic distribution of chromatin states in the `K562_1` dataset. The choice of the colors is arbitrary.

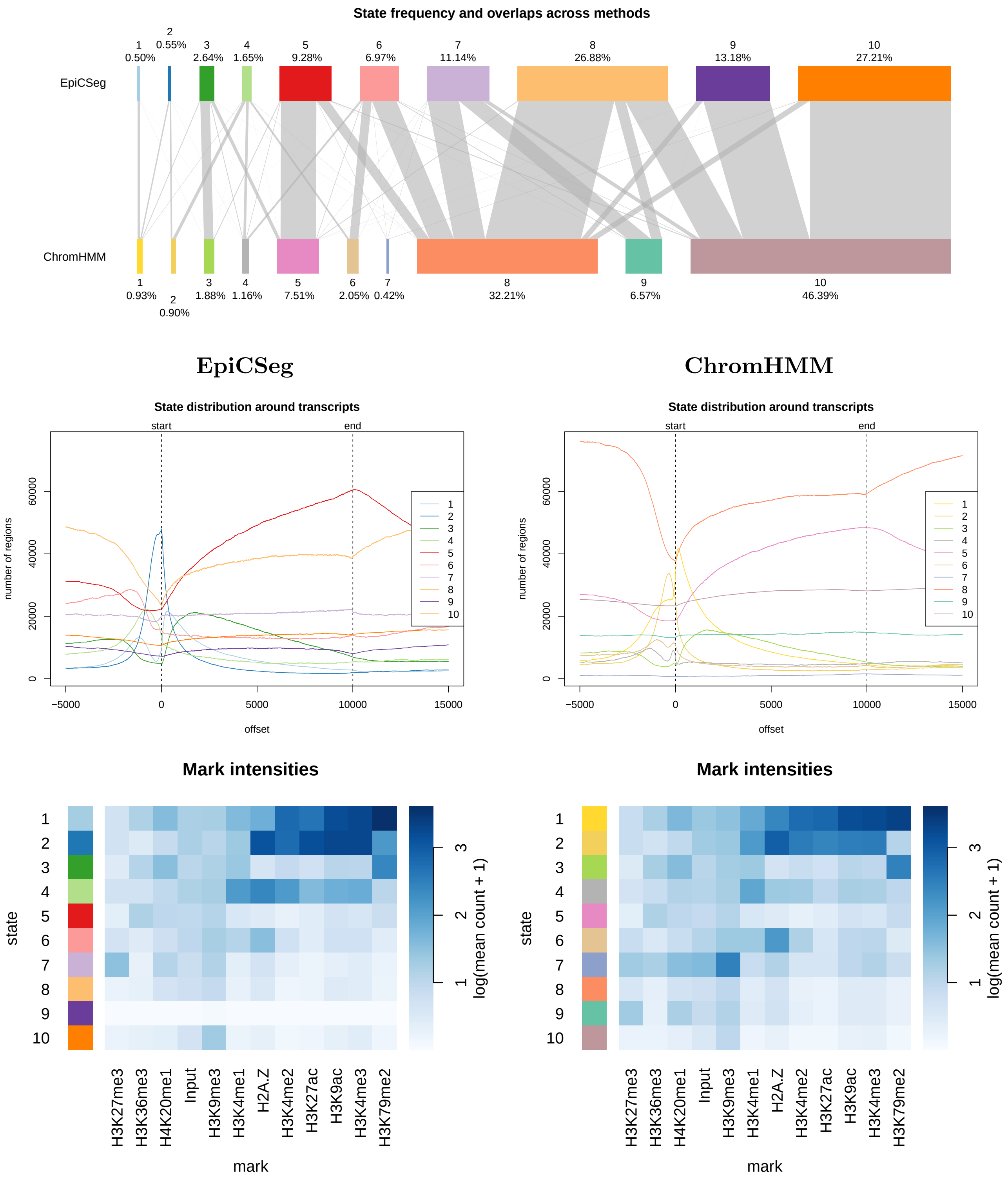

Figure 15: Genomic distribution of chromatin states in the `K562_2` dataset. The choice of the colors is arbitrary.

References

[1] Richard Durbin, Sean R. Eddy, Anders Krogh, and Graeme Mitchison. *Biological Sequence Analysis: Probabilistic Models of Proteins and Nucleic Acids*. Cambridge University Press, 2005.

[2] Bruce Levin and James Reeds. Compound multinomial likelihood functions are unimodal: Proof of a conjecture of IJ Good. *The Annals of Statistics*, 5(1):79–87, 1977.

[3] Roderick J A Little and Donald B Rubin. *Statistical analysis with missing data*. John Wiley & Sons, Inc., 1986.
